# Rat Calvarial Bone Regeneration by 3D-Printed β-Tricalcium Phosphate Incorporating MicroRNA-200c

**DOI:** 10.1021/acsbiomaterials.0c01756

**Published:** 2021-08-26

**Authors:** Matthew T. Remy, Adil Akkouch, Li He, Steven Eliason, Mason E. Sweat, Tadkamol Krongbaramee, Fan Fei, Fang Qian, Brad A. Amendt, Xuan Song, Liu Hong

**Affiliations:** † Iowa Institute for Oral Health Research, College of Dentistry, The University of Iowa, Iowa City, Iowa 52242, United States; ‡ Department of Industrial and Systems Engineering, College of Engineering, The University of Iowa, Iowa City, Iowa 52242, United States; § Department of Anatomy and Cell Biology, Carver College of Medicine, The University of Iowa, Iowa City, Iowa 52242, United States; ∥ Center for Craniofacial Anomalies Research, Carver College of Medicine, The University of Iowa, Iowa City, Iowa 52242, United States

**Keywords:** 3D printing, β-TCP, miR-200c, bone regeneration, calvarial defect

## Abstract

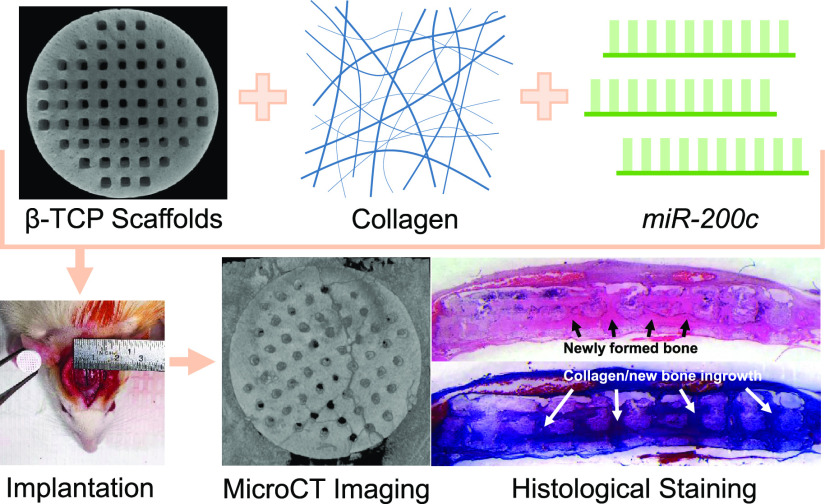

Advanced fabrication methods for bone grafts designed to match defect sites that combine biodegradable, osteoconductive materials with potent, osteoinductive biologics would significantly impact the clinical treatment of large bone defects. In this study, we engineered synthetic bone grafts using a hybrid approach that combined three-dimensional (3D-)printed biodegradable, osteoconductive β-tricalcium phosphate (β-TCP) with osteoinductive microRNA(miR)-200c. 3D-printed β-TCP scaffolds were fabricated utilizing a suspension-enclosing projection-stereolithography (SEPS) process to produce constructs with reproducible microarchitectures that enhanced the osteoconductive properties of β-TCP. Collagen coating on 3D-printed β-TCP scaffolds slowed the release of plasmid DNA encoding *miR-200c* compared to noncoated constructs. 3D-printed β-TCP scaffolds coated with *miR-200c*-incorporated collagen increased the transfection efficiency of *miR-200c* of both rat and human BMSCs and additionally increased osteogenic differentiation of hBMSCs *in vitro*. Furthermore, *miR-200c*-incorporated scaffolds significantly enhanced bone regeneration in critical-sized rat calvarial defects. These results strongly indicate that bone grafts combining SEPS 3D-printed osteoconductive biomaterial-based scaffolds with osteoinductive miR-200c can be used as superior bone substitutes for the clinical treatment of large bone defects.

## 1 Introduction

The restoration of large bone defects after traumatic injuries, tumor resections, and congenital diseases represents complex orthopedic and plastic surgical problems that often necessitate bone grafting.^1−3^ The outcomes of bone defect restoration are further complicated by factors, such as advanced age, severity of injury, degree of soft tissue damage, and comorbidities including osteoporosis and diabetes.^4^ While autografts are the current gold standard for treating bone defects, supply limitations and donor-site morbidity restrict their therapeutic application.^5^ Allografts may be used alternatively and represent nearly one-third of all bone grafts in North America. Yet, their clinical use is hindered by issues with immunological rejection and the risk of disease transfer.^6^ Moreover, the geometric irregularities of bone defects make graft-defect matching extremely challenging.^7^

Tissue engineering has emerged as a promising technology that combines biomaterials, stem cells, and bioactive molecules to create synthetic bone tissue substitutes in an attempt to surmount the need for natural bone grafts. A successful tissue-engineered bone graft capable of use for clinical application demands safe and biodegradable constructs retaining strong osteoconductive and osteoinductive capabilities that can practically restore relatively large-sized bone defects. A variety of prior works have designed tissue-engineered (TE) bone constructs using an abundance of materials and scaffold fabrication methods. Yet, inefficient osteoinductive agents and insufficient fabrication methods have prevented the clinical translation of these TE grafts. In addition, previous bone regeneration studies have heavily relied on the use of osteogenic growth factors, including recombinant human bone morphogenetic proteins (rhBMP-2, rhBMP-7),^8−13^ parathyroid hormone (PTH),^14,15^ and others, to enhance bone regeneration in synthetic bone grafts.^16−18^ However, recombinant growth factors are expensive and unstable, and the short half-life of these agents requires the administration of supraphysiological doses, which have been linked to a growing and well-documented side effect profile including tumorigenesis, postoperative inflammation and associated adverse effects, ectopic bone formation, osteoclast-mediated bone resorption, and inappropriate adipogenesis.^17,19−24^

Synthetically engineered bone grafts for clinical application require the exploitation of efficient osteoinductive agents in combination with effective scaffolding materials. TE bone grafts necessitate a scaffold that not only has an optimized internal microarchitecture that promotes cell migration, differentiation, and nutrient infiltration but also is versatile in shape and size to accurately fill the bone defects.^25^ Advancements in three-dimensional (3D) printing technologies have provided a promising tool to significantly transform scaffold fabrication techniques and expand the capabilities of modern bone tissue engineering. In addition to the precise design of a porous microarchitecture that optimizes osteoconductive capacities, 3D printing of synthetic bone scaffolds allows for the design of custom grafts, which provides patient-treatment specificity currently unavailable with natural grafts.^26,27^ Among a breadth of different scaffold fabrication techniques and materials previously utilized in bone regeneration strategies,^17,28−37^ both hydroxyapatite and β-TCP are biocompatible materials with similar chemical structures to the native bone that provide practical osteoconductive activities for bone regeneration. Furthermore, β-TCP has a superior osteoconductivity and is more easily remodeled after implantation due to its relatively high rate of biodegradation.^38−40^ Additionally, β-TCP-based scaffolds can provide more initial mechanical support as a bone graft compared to other mechanically weaker alternatives, such as biopolymers and extracellular matrix-based scaffolds.^41−44^ Yet, it is difficult to generate sufficient bone to restore large defects using β-TCP alone due to its limited osteoinductive properties.^23,38,39,45−47^

MicroRNAs (miRs) are small noncoding RNAs that post-transcriptionally regulate physiological and pathophysiological pathways through directly targeting the 3′UTRs of specific messenger RNA to cause degradation and/or translational repression.^48^ miRs play crucial roles in bone development and metabolisms and have recently been explored for their therapeutic potential in bone healing and regeneration. *miR-200c*, a member of the miR-200 family, plays critical roles in anticancer by inhibiting epithelial-to-mesenchymal transition (EMT) in cancer initiation and metastases.^49−52^*miR-200c* also executes a strong anti-inflammatory function in inflammation by directly targeting several proinflammatory cytokines and mediators.^53−56^ During osteogenic differentiation, *miR-200c* has been reported to directly target *Noggin,*(57,58) an antagonist of BMP signaling, and stem cell transcription factors, including *Klf4* and *Sox2*.^56^ Our previous studies demonstrated that *miR-200c* effectively increases osteogenic differentiation of human bone marrow mesenchymal stem cells (hBMSCs) and *miR-200c* incorporation into collagen sponges effectively promotes bone regeneration by upregulating Wnt signal activities.^53^

Therefore, the characteristics of this potent osteogenic agent strongly support *miR-200c* as a novel osteoinductive factor that may critically impact clinical bone regeneration as a safe and effective biological alternative to the insufficient current and traditional osteoinductive therapeutics.

In this study, for the first time, we fabricated an engineered bone graft using a hybrid approach that combines osteoconductive 3D-printed β-TCP scaffolds and osteoinductive *miR-200c*. The 3D-printed β-TCP was fabricated directly from a computer-aided design (CAD) model using an advanced stereolithography (SLA)-based additive manufacturing (AM) process. Collagen type-I was incorporated with plasmid DNA (pDNA) encoding *miR-200c* and coated onto the 3D-printed β-TCP scaffolds to investigate the retention of pDNA to the 3D-printed constructs and the influence of the *miR-200c*-incorporated collagen coatings on transfection efficiency and ultimately bone regeneration. We observed that coating β-TCP scaffolds with collagen incorporating *miR-200c* increased the retention of *miR-200c* and that *miR-200c*-incorporated collagen-coated β-TCP constructs effectively increased *miR-200c* expression in both rat and human bone marrow mesenchymal stem cells (BMSCs) while additionally enhancing osteogenic differentiation of hBMSCs *in vitro*. Furthermore, *miR-200c*-incorporated collagen-coated β-TCP scaffolds significantly promoted *in vivo* bone regeneration in a rat model of critical-sized calvarial defects. These data strongly indicate that the innovative approach by incorporating *miR-200c* into 3D-printed bone grafts may critically impact the development of clinically relevant synthetic bone grafts for treating challenging patient-specific bone defects.

## 2 Materials and Methods

### 2.1 Preparation and Characterization of 3D-Printed β-TCP Scaffolds

**Figure 1:**
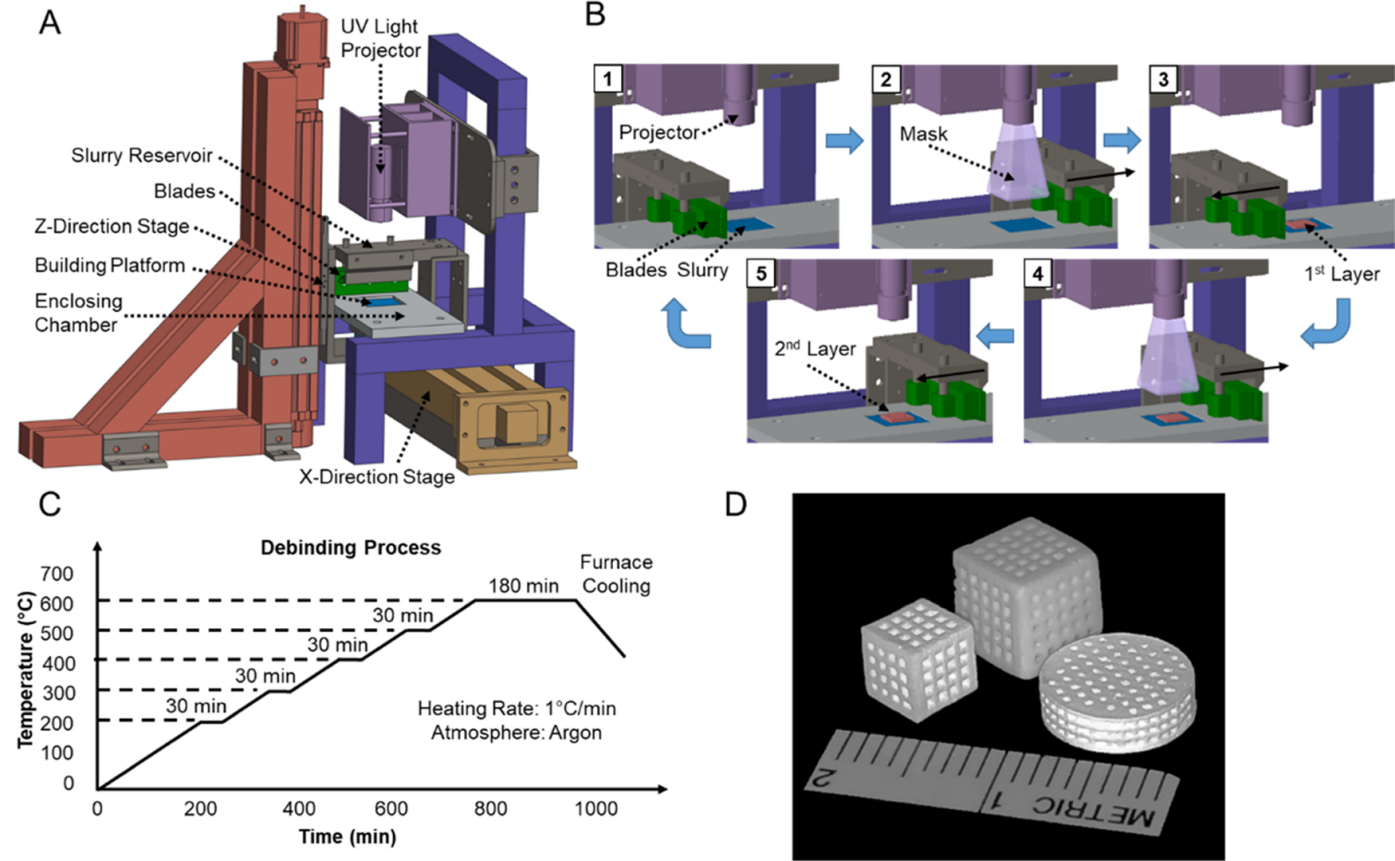
Diagram of β-TCP scaffold fabrication using SEPS. (A) 3D printer setup. (B) Layer-by-layer fabrication process. (C) Postprocess procedural debinding protocol to remove excess binder resin solution from the β-TCP-printed component. (D) Photographs of 3D-printed β-TCP scaffolds in various shapes and sizes.

**Figure 2:**
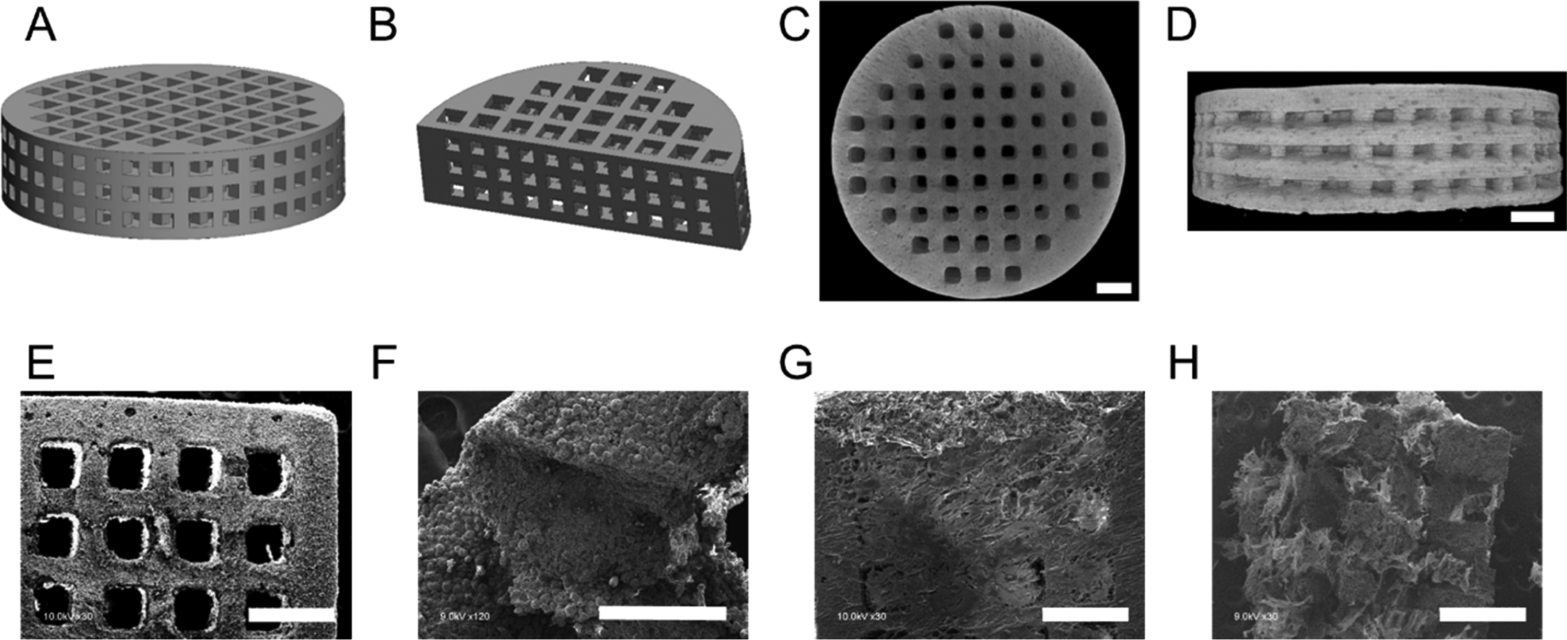
Characterization of 3D-printed β-TCP scaffolds. (A, B) CAD files of 3D-printed β-TCP scaffolds. (C, D) μCT images of 3D-printed β-TCP scaffold architectures and porosities. (E–H) SEM images of noncoated (E, F) and collagen-coated (G, H) surfaces and cross-sectional views. Scale bars: 1 mm (C, D, E, G, H) and 400 μm (F).

3D-printed β-TCP scaffolds were fabricated utilizing a support-free suspension-enclosing projection-stereolithography (SEPS) process.^59,60^ Different from standard SLA principles, SEPS creates ceramic parts by completely enclosing the manufactured components in a high-yield-stress slurry during the entirety of the fabrication process (Figure 1A,B). Slurry materials were prepared by mixing β-TCP particles (*D*(50) = 35.5 μm; Ceramisys Ltd., Sheffield, England) and a clear photopolymer resin (FLGPCL02; Formlabs, Somerville, MA) at concentrations of 40 and 60 wt%, respectively. A digital micromirror device (DMD; Texas Instruments, Dallas, TX) was used to photocure each layer of the fabricated pieces *via* mask image projection from a 405 nm ultraviolet light (UV) source (Figure 1B-2). Each layer was exposed to 15 s of UV light to induce photopolymerization, resulting in printed layers of 100 μm thickness.

Postprocessing of β-TCP scaffolds included ultrasonic cleaning, debinding, sintering, and sterilization processes. Ultrasonic cleaning was utilized to remove any uncured residual resin or unbound β-TCP particles, which involved placing the manufactured components in 99% ethanol and then placing these materials into an ultrasonic cleaning machine for washing. β-TCP scaffolds were washed in the ultrasonic cleaning machine five separate times with each wash lasting 4 min. A debinding process was utilized to remove the cured resin material from fabricated pieces, which consisted of slowly increasing the applied temperature at a rate of 1 °C/min until reaching 600 °C. After being held at 600 °C for 3 h, the temperature was decreased to room temperature at a rate of 3 °C/min (Figure 1C). After debinding, β-TCP components were further densified through sintering at 1250 °C (heating rate 8 °C/min, holding time 3 h). The sintered scaffolds were then sterilized *via* autoclave prior to utilization in the *in vitro* or *in vivo* experiments. The autoclave sterilization process consisted of exposing the sintered β-TCP scaffolds to a temperature and pressure of 121 °C and 14.2 PSI, respectively, for 20 min, followed by an hour of drying period.

Characterization of the 3D-printed β-TCP scaffolds was performed to analyze dimensional and material properties, integrity, and reproducibility between prints. A total of six scaffolds were evaluated and quantified (*n* = 6). 3D-printed β-TCP scaffolds were assessed for volume and weight using a digital caliper (Moock Digital Caliper; Shenzhen Moock Technology Co., Ltd., Guangdong, China) and scale (Balance XSR205DU; Mettler Toledo, Leicester, England), respectively. To assess the structural architectures and internal porosities of the 3D-printed β-TCP scaffolds, the constructs were analyzed *via* high-resolution microcomputed tomography (μCT) (Skyscan model 1272; Bruker, Kontich, Belgium) using a voltage of 70 kV, a current of 142 μA, a rotation step of 0.4, a 0.5 mm Al filter, and an image pixel size of 10 μm. Reconstruction of 3D virtual models of scanned β-TCP scaffolds was performed using NRecon (NRecon software version 1.6.10.2; Micro Photonics Inc., Allentown, PA). CTvox (CTvox software version 3.3; Bruker, Kontich, Belgium) was utilized to create a 3D volume rendering and representative 3D images of the scaffolds. Measurements for the 3D-printed scaffolds including mean scaffold thickness, diameter, strut length, pore size, and porosity were measured and quantified from the reconstructed μCT images using ImageJ software (National Institute of Health).

### 2.2 Development of Hybrid 3D-Printed Scaffolds Incorporating *miR-200c*

Bone grafts of 3D-printed scaffolds incorporating *miR-200c* were prepared by coating 3D-printed β-TCP scaffolds with collagen type-I-containing plasmid DNA (pDNA) encoding *miR-200c* at different concentrations. A total of six groups of treated scaffolds were developed for scaffold visualization and assessment under *in vitro* culture conditions. These treatment groups included (1) β-TCP scaffold alone, (2) β-TCP scaffold coated with collagen alone, (3) β-TCP scaffold coated with collagen incorporating empty vector (EV) (5 μg/scaffold), (4) β-TCP scaffold soaked with pDNA encoding *miR-200c* solution (5 μg/scaffold), (5) β-TCP scaffold coated with collagen incorporating pDNA encoding *miR-200c* (1 μg/scaffold), and (6) β-TCP scaffold coated with collagen incorporating pDNA encoding *miR-200c* (5 μg/scaffold). The pDNA encoding *miR-200c* and empty vector (EV) as control were prepared according to our previous studies.^53,56^ A total of 50 μL sterilized collagen type-I (Corning, Bedford, MA) at 3 mg/mL containing different doses of pDNA encoding *miR-200c* or EV were loaded at the top of the autoclave-sterilized 3D-printed β-TCP scaffolds and allowed to disperse down into the scaffold interior. This amount of collagen solution infiltrated the whole scaffolds without overflow. The treated constructs were subsequently frozen at −80 °C overnight and then lyophilized for 48 h using a freeze dryer (Virtis Advantage Plus; SP Industries, Gardiner, NY). Field-emission scanning electron microscopy (FE-SEM; Hitachi S-4800, Japan) operating at a 10 kV accelerating voltage was utilized to observe the surface morphology of noncoated and collagen-coated hybrid scaffolds. Samples were dried under vacuum overnight and sputter-coated with gold prior to SEM imaging (K550 Emitech Sputter Coater; Electron Microscopy Services/Quorum, Hatfield, PA). Both surface and cross-sectional images were acquired at different magnifications using SEM to observe collagen network distribution across and within the scaffolds.

### 2.3 Release of pDNA Encoding *miR-200c* from Noncoated and Collagen-Coated β-TCP Scaffolds

β-TCP scaffolds were 3D-printed, and three scaffold treatment groups were prepared under the same conditions as previously described to evaluate the release of pDNA encoding *miR-200c* from the scaffolds. These study groups included (1) β-TCP scaffold soaked with pDNA encoding *miR-200c* solution (5 μg/scaffold), (2) β-TCP scaffold coated with collagen incorporating pDNA encoding *miR-200c* (1 μg/scaffold), and (3) β-TCP scaffold coated with collagen incorporating pDNA encoding *miR-200c* (5 μg/scaffold). Treated scaffolds (*n* = 3/condition) were placed into individual wells in a sterile 12-well cell culture plate, and each well containing a scaffold was filled with 750 μL of sterile phosphate-buffered saline (PBS). The cell culture plate containing the treated scaffolds was placed on a shaker (Stovall Life Science Inc., Belly Dancer Shaker Orbital Platform Shaker; Thermo Fisher Scientific, Waltham, MA) to continuously shake at 100 rpm and room temperature for the duration of the release study. The concentration of pDNA released from the treated scaffolds was quantified using the NanoDrop One Microvolume UV–vis Spectrophotometers (Thermo Fisher Scientific, Waltham, MA) at distinct time points. pDNA concentration for each scaffold was measured in triplicate.

### 2.4 Determining Osteoinductive Capabilities of the β-TCP Scaffolds Incorporating *miR-200c* on Rat and Human BMSCs

Rat BMSCs (rBMSCs) were isolated from the femurs and tibias of 12 week old Sprague Dawley rats (Charles River Laboratories, Wilmington, MA) using a standardized isolation protocol.^61^ rBMSCs were cultured and expanded in Dulbecco’s modified Eagle’s medium (DMEM) supplemented with 10% fetal bovine serum (FBS) and 1% penicillin–streptomycin (PS) (Life Technologies, Grand Island, NY). rBMSCs in the supplemented DMEM media (DMEM Complete Medium) were cultured at 37 °C, 5% CO_2_. rBMSCs were cultured as a monolayer of cells either in a 6-well plate or in a 24-well plate on treated β-TCP scaffolds (scaffold treatments described in Section 2.2). For the rBMSC:β-TCP scaffold culture, in a cell culture plate (CELLSTAR 24-Well Plate; Greiner Bio-One, Monroe, NC), a 50 μL total cell suspension containing 5 × 10^5^ rBMSCs at passage 2 was added dropwise onto each scaffold in each of the six treatment groups and allowed to attach for 1 h. Complete DMEM medium was added to the plates containing the cell-loaded scaffolds, and the plates were then placed in an incubator to culture.

Primary human BMSCs (hBMSCs; StemCells, Newark, CA) were cultured and expanded with completed minimum essential medium (MEM-α) supplemented with 10% FBS and 1% PS. In a cell culture plate, a total of 50 μL cell suspension containing 5 × 10^5^ hBMSCs at passages 3–5 were added dropwise onto each scaffold in each of the six treatment groups and allowed to attach for 1 h. Complete DMEM medium was added to the plates containing the cell-loaded scaffolds, and the plates were then placed into an incubator to culture. To visualize the cell distribution and localization, the hBMSC-loaded scaffolds were incubated in DAPI (4′,6-diamidino-2-phenylindole, dihydrochloride) stain solution for 5 min at room temperature according to the manufacture’s protocol (Invitrogen, Carlsbad, CA). After DAPI incubation, the scaffolds were imaged to observe cell distribution on the scaffolds. DAPI fluorescence, with excitation/emission wavelengths at 350 and 470 nm, was observed using a fluorescent microscope (ECLIPSE Ts2-FL/Ts2; Nikon, Tokyo, Japan) utilizing the DAPI filter, and images were captured at 4× and 10× magnifications. To investigate the ultrastructure of the hBMSCs cultured on the *miR-200c*-loaded 3D-printed β-TCP scaffolds, the specimens were first rinsed with PBS, fixed in 4% paraformaldehyde for 15 min, and then washed with distilled water. Dehydration was performed in a series of ethanol solutions of increasing concentrations (50, 70, 90, and twice at 100%). The dehydrated specimens were kept overnight in a vacuum oven at room temperature. Specimens were sputter-coated with gold and examined with a field-emission scanning electron microscopy (FE-SEM; Hitachi S-4800, Japan) operating at a 10 kV accelerating voltage.

To evaluate cellular uptake of *miR-200c* and the *miR-200c* transfection efficiency in rBMSCs, rBMSC-seeded scaffolds, and hBMSC-seeded constructs, the expression of *miR-200c* in rBMSCs and hBMSCs was evaluated using quantitative real-time polymerase chain reaction (qRT-PCR). rBMSC monolayers and scaffolds seeded with either rBMSCs or hBMSCs were cultured in DMEM completed medium and analyzed at different time points to assess the cellular uptake of *miR-200c* and *miR-200c* transfection efficiency across each cell source and culture system (each treatment performed using technical triplicates). For the qRT-PCR analyses, the total cellular RNA from either the rBMSCs or hBMSCs was extracted using a miRNeasy Mini Kit (Qiagen, Valencia, CA). The concentration and purity of total RNA were quantified using the NanoDrop One Microvolume UV–vis Spectrophotometers (Thermo Fisher Scientific, Waltham, MA) and verified using gel analysis. *miR-200c* expression was measured using the mirScript II reverse transcription kit and the mirScript SYBR Green PCR Kit (Qiagen, Valencia, CA) and normalized to glyceraldehyde 3-phosphate dehydrogenase (*GAPDH*), an internal control for human cells, *via* a comparative Ct (ΔΔCt) method. The primer sequence for GAPDH can be found in Table 1.

**Table 1:** Primer Sequences Used for *In Vitro* qRT-PCR Analysis

gene	forward primer	reverse primer
*GAPDH*	5′ TGTGGGCATCAATGGATTTGG 3′	5′ ACACCATGTATTCCGGGTCAAT 3′
*Runx2*	5′ TGGTTACTGTCATGGCGGGTA 3′	5′ TCTCAGATCGTTGAACCTTGCTA 3′
*OCN*	5′ CACTCCTCGCCCTATTGGC 3′	5′ CCCTCCTGCTTGGACACAAAG 3′
*OPG*	5′ GCTTGAAACATAGGAGCTG 3′	5′ GTTTACTTTGGTGCCAGG 3′

### 2.5 Quantitative Osteogenic Gene Analysis

To examine the effects of *miR-200c* on osteogenic differentiation of hBMSC-seeded scaffolds *in vitro*, the mRNAs of osteogenic biomarkers, including runt-related transcription factor 2 (*Runx2*), osteocalcin (*OCN*), and osteoprotegerin (*OPG*), were evaluated using qRT-PCR. Treated scaffolds were cultured in DMEM completed medium, and osteogenic gene expression was assessed *via* qRT-PCR at distinct time points (each treatment performed using technical triplicates). Total cellular RNA from hBMSCs on the treated constructs was extracted, quantified, and verified as previously completed to assess for *miR-200c* expression *via* qRT-PCR. To measure the mRNA expression of osteogenic markers using qRT-PCR, a total of 1 μg of RNA was reverse-transcribed using the PrimeScript reagent kit (Takara Bio Inc., Kusatsu, Shiga, Japan). Expression of *Runx2* and *OCN* was performed on a CFX Connect (Bio-Rad, Hercules, CA) using the SYBER Premix Ex Taq II Kit (Takara Bio Inc., Kusatsu, Shiga, Japan). Gene expression was calculated and normalized to *GAPDHvia* a comparative Ct (ΔΔCt) method. The primer sequences for *Runx2*, *OCN*, *OPG*, and *GAPDH* can be found in Table 1.

### 2.6 *In Vivo* Bone Regeneration by *miR-200c*-Loaded 3D-Printed β-TCP Scaffolds

All *in vivo* animal experiments were performed under the approval of the Office of Animal Resources at the University of Iowa. The surgical protocols were followed by the policies and guidelines provided by the Institutional Animal Care and Use Committee, and all animal surgeries were performed under sterile conditions. Treated scaffolds for the *in vivo* studies were sterilized and loaded with collagen and pDNA under the same conditions and in the same sterile environment as previously described for the *in vitro* studies. The scaffolds incorporated with different pDNAs encoding *miR-200c* and EV were implanted into 12 week old male Sprague Dawley rats (Charles River Laboratories, Wilmington, MA). Under general anesthesia, using ketamine/xylazine, a mid-skin incision was made in the nasofrontal area to the external occipital protuberance on the rats. A single, 9 mm diameter full-thickness defect was generated on the rat parietal bones. A total of six groups of treated scaffolds were implanted into critical-sized defects in the rat skull to observe the regenerative effects of the *miR-200c*-incorporated hybrid constructs, including (1) β-TCP scaffold alone, (2) β-TCP scaffold coated with collagen alone, (3) β-TCP scaffold coated with collagen incorporating an empty vector (EV) (5 μg/scaffold), (4) β-TCP scaffold soaked with pDNA encoding *miR-200c* solution (5 μg/scaffold), (5) β-TCP scaffold coated with collagen incorporating pDNA encoding *miR-200c* (1 μg/scaffold), and (6) β-TCP scaffold coated with collagen incorporating pDNA encoding *miR-200c* (5 μg/scaffold). Each animal received one treated scaffold implant, and each treatment condition had five animals per group (*n* = 5). All of the treated scaffolds were frozen at −80 °C overnight and then lyophilized for 48 h prior to implantation. All surgical operations were completed under sterile conditions. Rats were euthanized after 4 weeks, and the implanted constructs were harvested. Bone formation from the differently treated implants was analyzed using microcomputed tomography (μCT) and histology.

### 2.7 μCT Imaging

μCT imaging was performed to evaluate new bone formation within the scaffolds at the defect site. Specimens were analyzed *via* μCT (Skyscan model 1272, Bruker, Kontich, Belgium) at a voltage of 70 kV, a current of 142 μA, a rotation step of 0.5 mm Al filter, and an image pixel size of 18 μm. Reconstruction of 3D virtual models was performed with NRecon software, as previously described. CTvox software was utilized to create a 3D volume rendering and representative 3D images of the defect and integrated implants, as previously described. The same μCT threshold was applied across all samples to ensure identical imaging parameters when comparing each scanned sample. Images for each sample were taken from the top-down to assess bone formation occurring in the vertical pore channels. Cross-sectional images were additionally taken spanning the diameter of the scaffolds in both *X* and *Y* directions to evaluate bone formation within the horizontal porous channels of the treated β-TCP scaffolds.

### 2.8 Histomorphometric Analysis of Bone Formation and Integration

After μCT imaging was completed, the explanted calvarial tissues were decalcified using a 15% ethylenediaminetetraacetic acid (EDTA) solution. Tissues were decalcified for 2 weeks, then rinsed in PBS, and dehydrated *via* treatment with an ethanol gradient. The decalcified samples were then cleared with xylene and embedded in paraffin for sectioning. The entire embedded sample, which included the defect site with the implanted treated β-TCP scaffold and the surrounding native bone tissue, was cut into 7 μm coronal sections and stained with hematoxylin and eosin (H&E) and Masson’s Trichrome stain using standard protocols. Representative sections were selected for staining and histomorphometric analysis at distinct intervals throughout the sample, starting from the middle of the sample and working outwards at an interval sampling distance of 0.5 mm (*n* = 5). At each sampling interval, a section was stained with H&E and another using Masson’s Trichrome stain. Corresponding images of the H&E and Masson’s Trichrome stained tissues were taken using an encoded stereo surgical microscope (Leica M125 C; Leica, IL) to examine the bone formation and integration of the implant with the surrounding native bone tissues. Histomorphometric analysis was conducted using ImageJ software to quantify new bone formation within the defect site, and these values are reported as an area percentage (bone area/total defect area, %) with standard deviations. To ensure that the histomorphometric analysis results using ImageJ were correct in identifying bone tissues and differentiating new bones from fibrous tissues, our results were confirmed by the University of Iowa pathology laboratory.

### 2.9 Statistical Analysis

Descriptive statistics were conducted for both *in vitro* and *in vivo* investigations. A one-way analysis of variance (ANOVA) with post hoc Tukey’s honestly significant difference (HSD) test was used to determine whether there was a significant difference between treatment groups for the *in vitro miR-200c* and osteogenic marker expression studies. For the *in vivo* study, a one-way ANOVA with post hoc Tukey’s HSD test was utilized to evaluate whether there were significant differences between the H&E stained sections across all six treatment groups. The Shapiro–Wilks test was also applied to verify the assumption of normality. All statistical tests completed for the *in vitro* and *in vivo* quantifications used a significance level of 0.05, and each graphic depicts mean values and associated standard deviations (SDs). Statistical analyses were performed using the statistical packages SAS System version 9.4 (SAS Institute Inc., Cary, NC) and GraphPad Prism (version 8.1.2; San Diego, CA).

## 3 Results

### 3.1 Fabrication and Characterization of 3D-Printed Collagen-Coated Hybrid Scaffolds

The 3D-printed β-TCP scaffolds were fabricated from CAD files using SEPS and were designed to have porous channels running from the top-down and through the sides of each scaffold, creating a lattice network with interconnected pores (Figure 2A,B). The 3D-printed scaffolds were evaluated for mean pore size, porosity, and other dimensional parameters and material properties, and these are reported in Table 2. The SEPS fabricated scaffolds had an average diameter and thickness of 8.8 and 2.5 mm, respectively, with well-defined, reproducible porous channels running throughout the 3D-printed constructs (Figure 2C,D). The scaffolds had an average porosity of 44.16%, and each pore had an average diameter of 410 μm (Figure 2E). Cross-sectional cuts through the scaffold showed β-TCP particles evenly distributed within the interior struts of the scaffold (Figure 2F). In scaffolds with collagen coating, the surface topography and collagen distribution on the β-TCP scaffolds were observed using SEM imaging. Collagen-coated scaffolds demonstrated collagen network distribution across the surface of the construct (Figure 2G). Additionally, cross-sectional cuts through the scaffold showed that the fibrous collagen network was not only localized to the loading surface but was able to disperse through the entirety of the construct (Figure 2H).

**Table 2:** Dimensional Parameters and Material Properties for 3D-Printed β-TCP Scaffolds

dimensional parameters	mean (SD)	material properties	mean (SD)
pore diameter (μm)	410.084 (47.151)	volume (mm^3^)	152.203 (0.740)
strut diameter (μm)	393.088 (43.317)	weight (mg)	132.966 (4.704)
diameter (mm)	8.799 (0.021)	density (g/cm^3^)	0.867 (0.030)
thickness (mm)	2.503 (0.008)	porosity (%)	44.156 (0.700)

### 3.2 Coating of *miR-200c*-Loaded Collagen Facilitates hBMSC Attachment to 3D-Printed β-TCP Scaffolds

**Figure 3:**
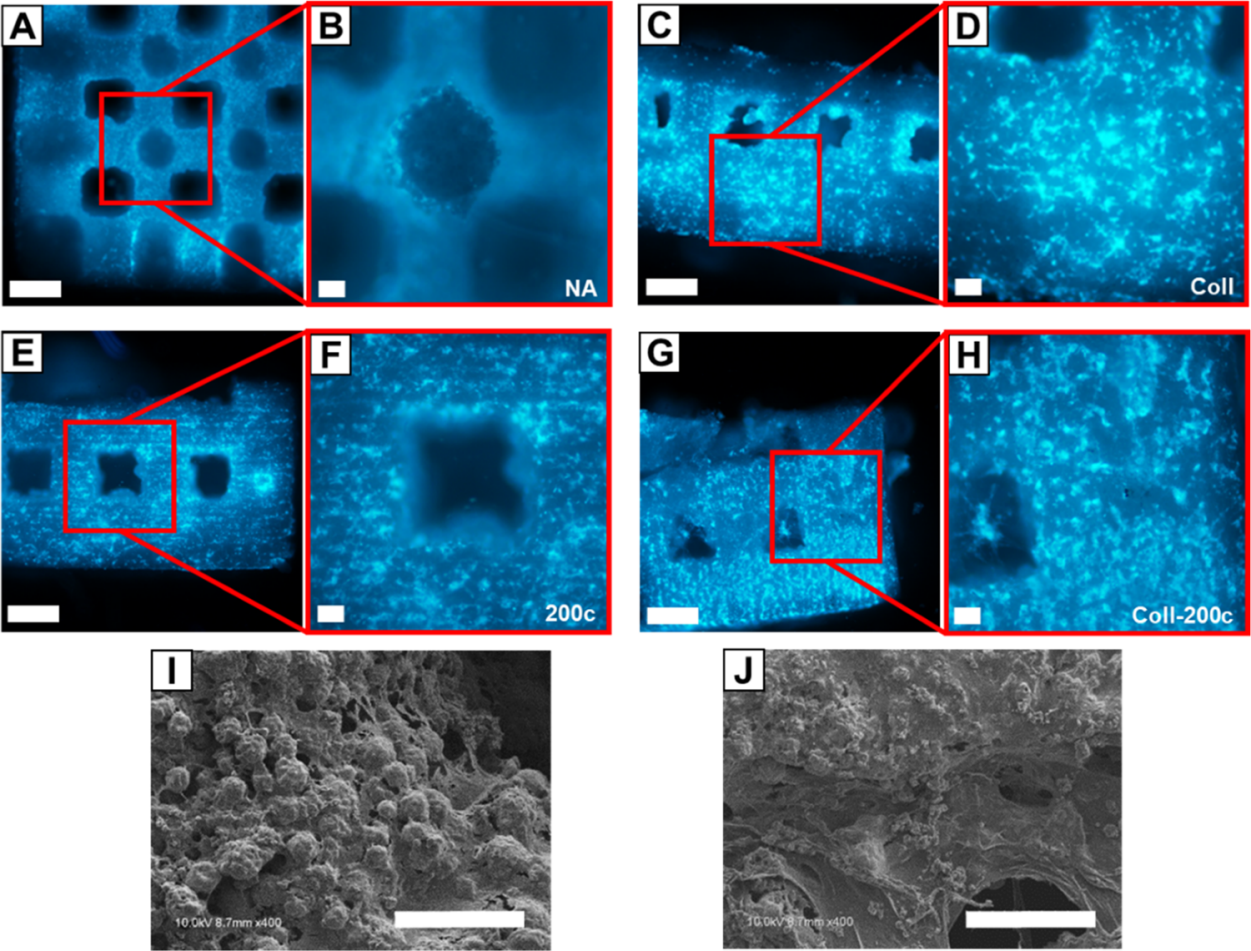
Images of hBMSC distribution on β-TCP scaffolds. (A–H) DAPI nuclear-stained images of noncoated β-TCP scaffolds (A, B); collagen-coated β-TCP (C, D); noncoated β-TCP with incorporated *miR-200c* (E, F); collagen-coated, *miR-200c*-incorporated β-TCP (G, H). (I, J) SEM images of hBMSC attachment to collagen network and matrix production at the β-TCP scaffold surface. Scale bars: 500 μm (A, C, E, G) and 100 μm (B, D, F, H, I, J).

After hBMSCs were pipetted onto the top surface of the β-TCP scaffolds, we observed that the cells dispersed throughout the constructs. Figure 3 summarizes the distribution of the DAPI-stained hBMSCs 3 days after seeding into β-TCP scaffolds with different treatments. hBMSCs homogeneously distributed across the surfaces and interior portions of the β-TCP scaffolds. The distribution of hBMSCs was not affected by different treatment conditions and exhibited the same homogeneous cell distribution across the 3D-printed construct (Figure 3A–H). Under SEM imaging, the hBMSCs amply attached to and produced extracellular matrix across the surface of the β-TCP scaffolds (Figure 3I). Cross-sectional cuts through the scaffolds also demonstrated that the interconnected porous network allowed for cell infiltration, distribution, and matrix production across the entirety of the scaffold (Figure 3J).

### 3.3 Collagen Coatings Slow Release of pDNA Encoding *miR-200c* from β-TCP Scaffolds

**Figure 4:**
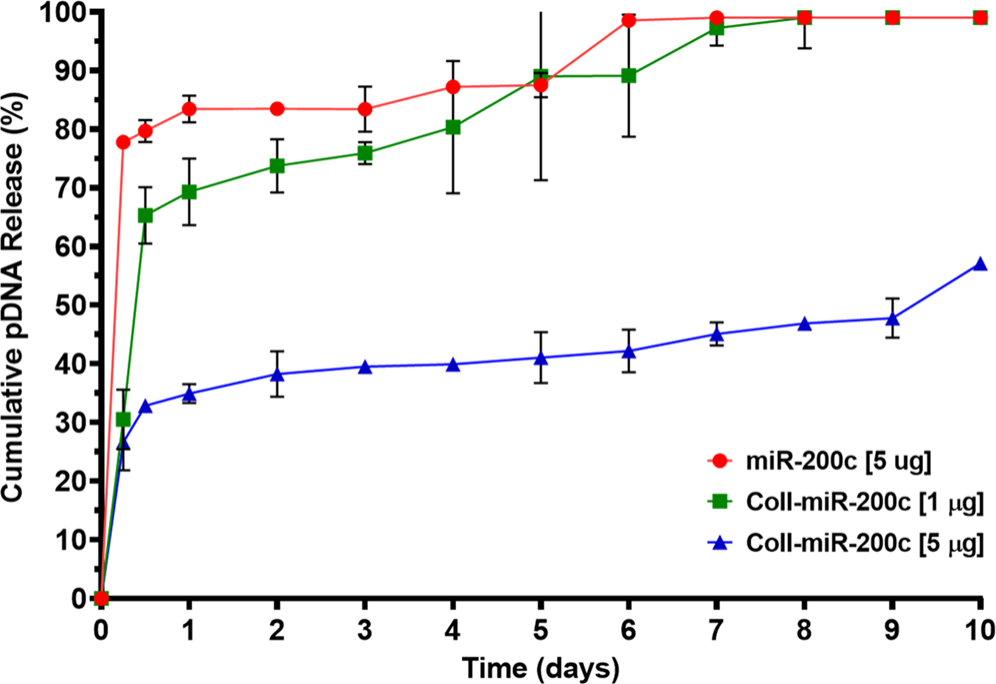
Collagen incorporating *miR-200c* slowed the release of *miR-200c* from β-TCP scaffolds. A 10 day cumulative release of pDNA encoding *miR-200c* from 3D-printed β-TCP scaffolds coated with or without collagen at different concentrations.

Over the 10 day period observed for pDNA release from β-TCP scaffolds coated with or without collagen at different concentrations, we found that collagen coatings on β-TCP scaffolds dramatically improved the retention of pDNA on β-TCP scaffolds in comparison to β-TCP scaffolds without collagen coating (Figure 4). We observed a burst release of pDNA encoding *miR-200c* for all scaffolds, regardless of coating, at the 6 h time point. However, we found that collagen-coated scaffolds, particularly the collagen-coated scaffolds incorporating *miR-200c* at 5 μg, demonstrated a sustained release function after the first 24 h of release. For the noncoated scaffolds, over 80% of incorporated *miR-200c* was released by 24 h, while the collagen-coated scaffolds, particularly the collagen-coated scaffolds incorporating 5 μg *miR-200c*, released less *miR-200c* (Coll-miR-200c [1 μg] 70%; Coll-miR-200c [5 μg]: 35%) after 24 h. After the first 24 h, scaffolds without collagen coating released pDNA at a higher rate for the remainder of the 10 day evaluation period in comparison to those coated with collagen incorporating 5 μg *miR-200c*. We also observed that the noncoated scaffolds released approximately 100% of incorporated *miR-200c* by day 6. For the collagen-coated scaffolds, the scaffolds incorporating 1 μg released 100% of incorporated *miR-200c* by day 8 and the scaffolds incorporating 5 μg released approximately 57% of incorporated *miR-200c* by day 10.

### 3.4 Enhanced *miR-200c* Expression and Osteogenic Differentiation of rBMSCs and β-TCP Scaffolds Seeded with Either rBMSCs or hBMSCs in *miR-200c*-Incorporated Scaffolds

**Figure 5:**
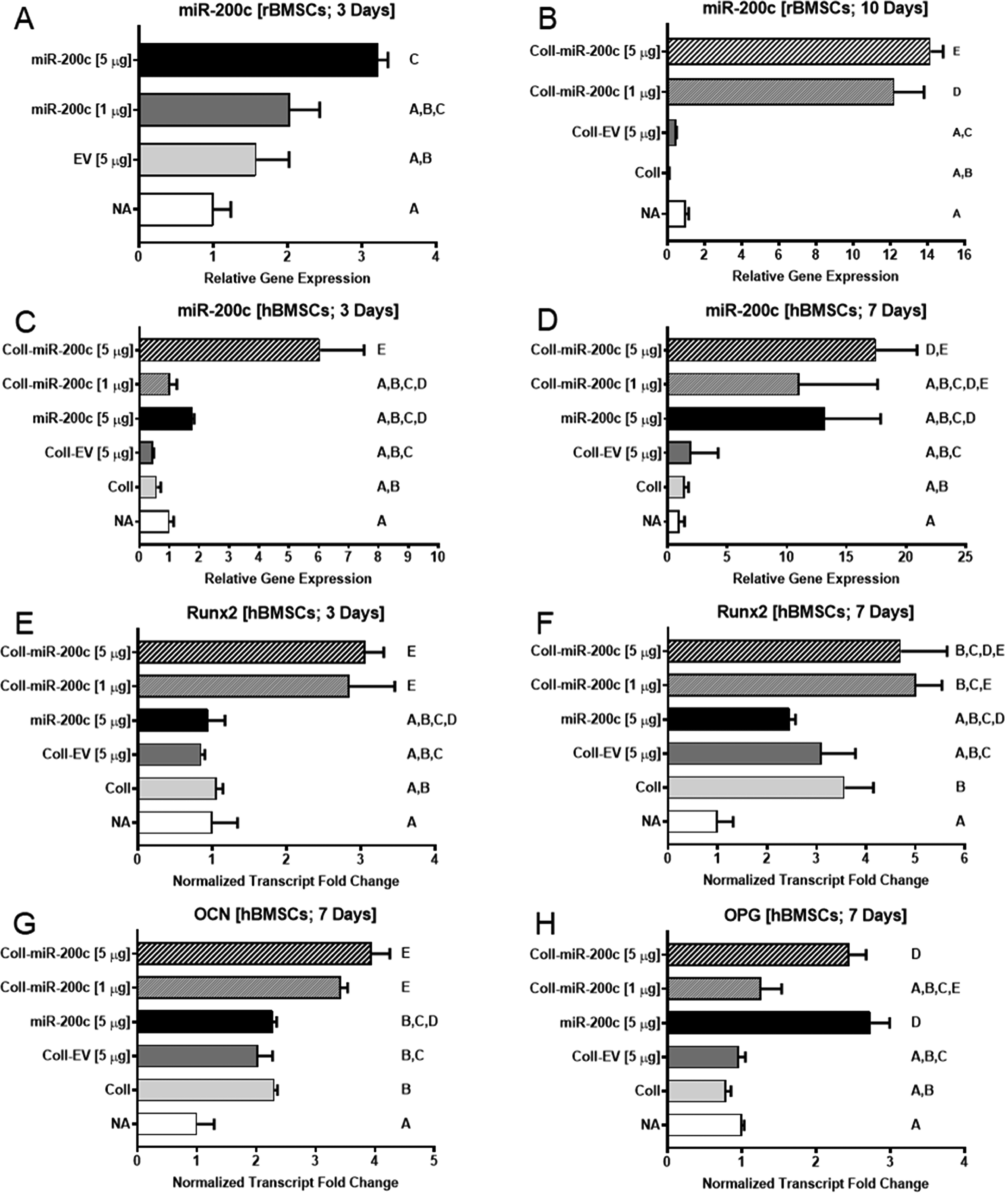
Collagen incorporating *miR-200c* increased *miR-200c* expression and osteogenic differentiation of rat and human BMSCs seeded on 3D-printed β-TCP scaffolds. (A) Relative expression levels of *miR-200c* from rBMSCs cultured as a monolayer for 3 days with different concentrations of pDNA encoding *miR-200c* or empty vector control. (B) Relative expression levels of *miR-200c* from rBMSCs 10 days after seeding onto β-TCP scaffolds with different treatments. (C, D) Relative expression levels of *miR-200c* from hBMSCs 3 days (C) and 7 days (D) after seeding onto β-TCP scaffolds with different treatments. (E, F) Normalized fold change of *Runx2* transcript from hBMSCs 3 days (E) and 7 days (F) after seeding. (G, H) Normalized fold change of *OCN* (G) and *OPG* (H) transcripts in hBMSCs 7 days after seeding onto β-TCP scaffolds with different treatments. Column means that do not share a letter are statistically significantly different using the post hoc Tukey’s HSD test (*p* < 0.05; performed in triplicate).

After transfecting rBMSCs cultured in a monolayer environment with either empty vector control plasmid or pDNA encoding *miR-200c* at different concentrations, we found that the rBMSCs transfected with high-concentration *miR-200c* plasmid significantly increased the expression of *miR-200c* compared to empty vector control and untreated rBMSCs (Figure 5A). We did not find a significant increase in expression of *miR-200c* for rBMSCs transfected as a monolayer with low-concentration *miR-200c* plasmid when compared to empty vector control and untreated rBMSCs. However, when rBMSCs were seeded on β-TCP scaffolds with different treatment conditions, scaffolds coated with collagen incorporating pDNA encoding *miR-200c* at both low and high concentrations statistically significantly increased the expression of *miR-200c* in contrast to β-TCP scaffolds coated with collagen or collagen incorporating empty vector control and untreated β-TCP scaffolds (Figure 5B). Moreover, β-TCP scaffolds coated with collagen incorporating pDNA encoding *miR-200c* at high concentrations statistically significantly increased *miR-200c* expression of rBMSCs to the greatest extent when compared to all other treatments (*p* < 0.05; performed in triplicate).

While β-TCP scaffolds loaded with pDNA solution encoding *miR-200c* at 5 μg/scaffold did not significantly increase *miR-200c* in the hBMSCs 3 days after cell seeding, the scaffolds coated with *miR-200c* incorporated collagen at the same *miR-200c* concentration significantly increased the expression of *miR-200c* compared to control groups with collagen alone and untreated scaffolds (Figure 5C). This indicated that pDNA of *miR-200c* was more effectively taken up by hBMSCs from the incorporation of miR-collagen-loaded scaffolds. Overexpression of *miR-200c* induced by *miR-200c-*incorporated collagen was kept in the scaffolds after 7 days (Figure 5D). pDNA solution encoding *miR-200c* at 5 μg/scaffold and *miR-200c-*incorporated collagen at 1 μg/per disc also increased after 7 days. We measured the osteogenic biomarkers, including *Runx2* and *OCN*, of hBMSCs seeded on β-TCP scaffolds after 3 and 7 days. After 3 days, the expression of *Runx2*, an early marker for osteogenic differentiation, was upregulated in the scaffolds coated with collagen incorporating *miR-200c* at different doses in comparison to scaffolds treated with only collagen or control scaffolds (Figure 5E). After 7 days, both transcripts of *Runx2* and *OCN* were significantly increased in the cells within the scaffolds treated with collagen incorporating *miR-200c* (Figure 5F,G). However, the osteogenic differentiation markers in the cells of the scaffolds treated with pDNA encoding *miR-200c* alone were hardly changed compared to the nontreated scaffolds and scaffolds treated with collagen alone. Furthermore, expression of *OPG* at 7 days was significantly increased in the cells within scaffolds treated with collagen incorporating *miR-200c* [5 μg] and those treated with pDNA encoding *miR-200c* [5 μg] alone, in comparison to collagen incorporating *miR-200c* [1 μg] and nontreated or collagen control scaffolds (Figure 5H).

### 3.5 Bone Regeneration Induced by Hybrid Scaffolds of 3D-Printed β-TCP Coated with Collagen Incorporating *miR-200c*

**Figure 6:**
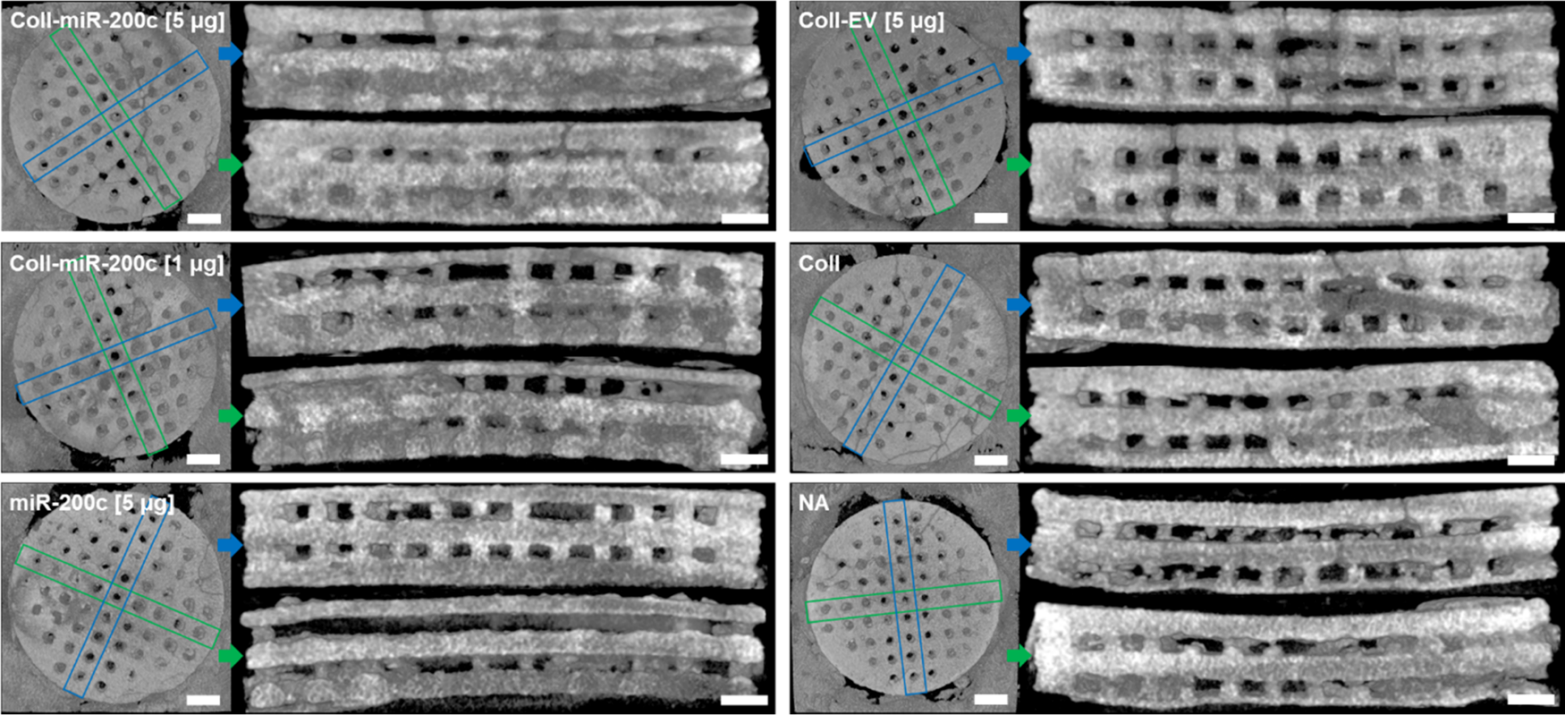
μCT images of bone regeneration induced by 3D-printed β-TCP scaffolds coated with collagen incorporating *miR-200c*. Representative μCT images of top and cross-sectional side views of explants 4 weeks postoperatively. Cross-sectional images were taken across the diameter of the β-TCP scaffolds in each direction (represented as blue or green boxes) to assess bone regeneration within each layer of the implanted constructs. Scale bars: 1 mm.

Figure 6 summarizes the μCT images of bone regeneration in the critical-sized defects 4 weeks after implantation of hybrid β-TCP scaffolds containing *miR-200c* or controls. Through μCT imaging, we were able to evaluate bone tissue growth within the porous scaffold channels by visually assessing X-ray beam attenuation, where low attenuation is representative of soft tissue development, while higher attenuation is associated with dense tissues, such as bone and calcifications. In the μCT images taken from the top-down through the scaffolds, we observed a higher X-ray beam attenuation in the filled-in vertical channels of the scaffolds coated with collagen incorporating *miR-200c* at both low- and high-concentration pDNA encoding *miR-200c*, while the majority of the porous channels in the β-TCP scaffold without treatment and the scaffolds coated with collagen incorporating EV remained transparent or with a significantly lower attenuation. While some channels of the scaffolds treated with the *miR-200c* solution also showed somewhat elevated X-ray beam attenuation, these channels were more transparent with a lower attenuation than the scaffolds treated with *miR-200c-*incorporated collagen. μCT images were additionally taken from the side of the implants to visualize the vertical layers of the scaffolds. We observed higher X-ray beam attenuation in the filled layers near to the dura mater in the implants without coating or coated with EV-incorporated collagen; however, the layers near to the periosteum kept transparency and low attenuation. Interestingly, we observed high X-ray beam attenuation in nearly the whole thickness of the scaffolds coated with collagen incorporating *miR-200c* at low and high concentrations of pDNA encoding *miR-200c* from dura mater to periosteum. In particular, cross-sectional images of the β-TCP scaffolds coated with collagen incorporating *miR-200c* at 5 μg demonstrated the highest X-ray beam attenuation compared to all other treatment groups, with nearly all pores filled through the full thickness of the β-TCP scaffolds.

**Figure 7:**
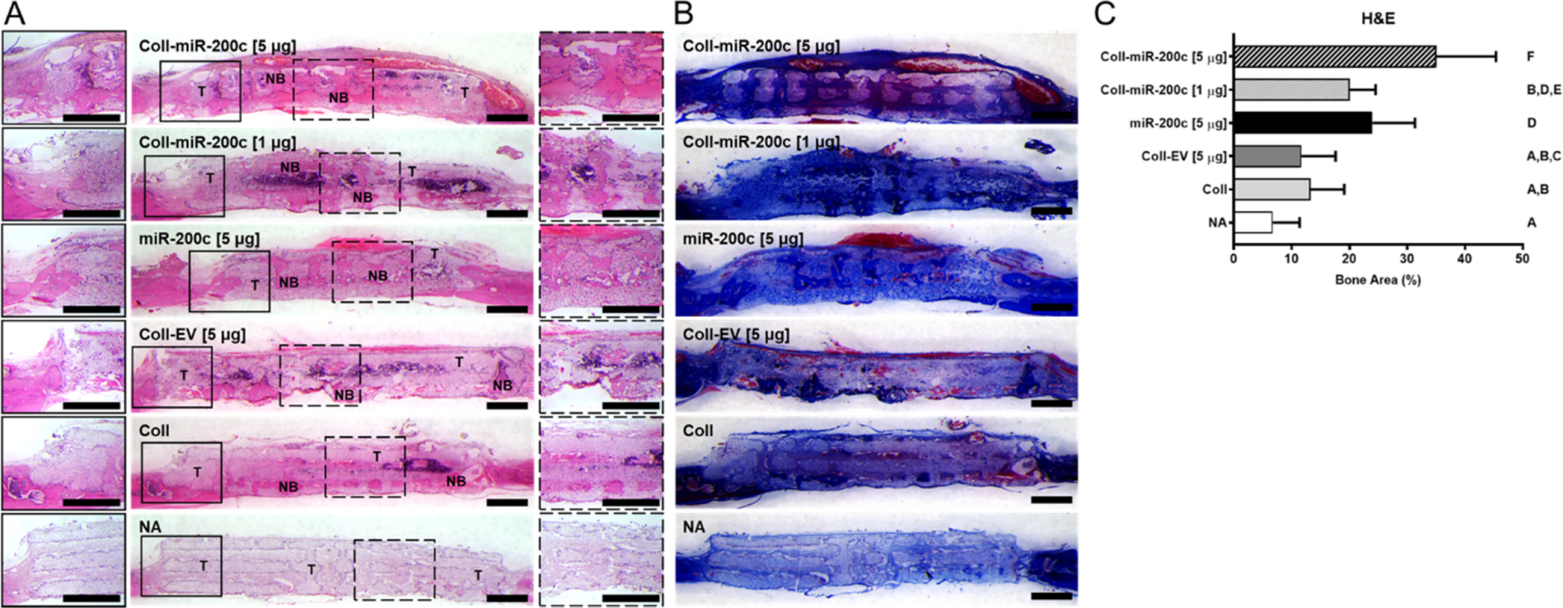
Histological analysis of new bone formation and integration of the implanted β-TCP scaffolds. (A, B) Microphotographs of cross sections of β-TCP scaffold incorporated with different *miR-200c* concentrations and controls 4 weeks after implantation: hematoxylin and eosin (H&E) (A) and Masson’s Trichrome (B) staining. (C) Histomorphometric analysis quantifying new bone formation in H&E stained β-TCP scaffold sections. Column means that do not share a letter are statistically significantly different using the post hoc Tukey’s HSD test (*p* < 0.05; *n* = 5). Scale bars: 1 mm. NB, new bone; T, β-TCP.

In the histological sections of the explanted calvarial tissues containing treated scaffolds stained with H&E (Figure 7A) and Masson’s Trichrome stains (Figure 7B), we observed a few scattered bone formations in the β-TCP scaffolds alone or those coated with collagen. However, new bone formation across the entirety of the scaffolds was found in the β-TCP scaffolds coated with collagen incorporating *miR-200c* at both low and high concentrations of pDNA encoding *miR-200c*. In particular, scaffolds coated with collagen incorporating *miR-200c* at 5 μg observed a statistically significant increase in bone formation compared to all other treatment groups (Figure 7C). We also observed that the scaffolds coated with collagen incorporating EV failed to induce bone formation comparable to β-TCP scaffolds treated with *miR-200c* solution without collagen coatings and β-TCP scaffolds coated with collagen incorporating *miR-200c* at both low and high *miR-200c* concentrations. The Masson’s Trichrome staining showed that the newly formed bone shared similar amounts of collagen as in natural bone. Additionally, the scaffolds treated with either collagen or *miR-200c* alone displayed bone tissue formation occurring on the periphery of the implant, directly adjacent to the native tissue, demonstrating that all β-TCP scaffolds integrated well with the surrounding native bone.

## 4 Discussion

There is a critical need to develop effective TE bone grafts to successfully clinically treat large bone defects, particularly those that utilize safe and efficient osteoinductive agents in combination with osteoconductive scaffolding materials. Complete bone regeneration is complex, and many pathophysiological conditions in patients, including aging, estrogen insufficiency, and radiation therapy after tumor resection, may impact endogenous osteogenic activities and regenerative capabilities.^25,62−64^ Therefore, exogenous osteogenic factors and osteoprogenitor cells are needed to effectively induce efficient bone regeneration.^22,65−67^ A hybrid approach designed to produce scaffolds with osteoconductive and -inductive activities may address these concerns and create synthetic bone grafts that overcome the deficiencies of current standard bone grafts. In this study, we have revealed, for the first time, that incorporation of osteoinductive *miR-200c* into collagen-coated, 3D-printed osteoconductive β-TCP effectively promotes rat and human BMSC transfection and increases hBMSC osteogenic differentiation and bone regeneration in a rat critical-sized calvarial defect model. The combination of 3D-printed osteoconductive β-TCP scaffolds and osteoinductive *miR-200c* significantly advances synthetic bone regeneration due to the incorporation of safer, yet potent osteoinductive biologics and improved fabrication methods.

A plethora of scaffold fabrication techniques for engineering bone substitutes have previously been investigated.^34,35,68−72^ Advances in scaffold design methodologies have led to state-of-the-art 3D printing technologies that allow for the precise control over pore size, geometry, and distribution, permitting the design of interconnected porous networks that facilitate cell attachment and increase mass transport of oxygen and nutrients throughout the construct.^6,18,30,45,68,73−79^ In particular, stereolithography (SLA) has been used to produce ceramic bone substitutes; however, this process requires the use of support features for overhanging or fragile parts and removal of these structures can introduce fracture tips and microcracks, which can propagate and weaken the construct.^72,80−84^ In overcoming these challenges, SEPS, an advanced SLA printing technique, has been developed to produce complex ceramic scaffolds with increased resolution, higher densities, and greater geometric fidelity.^60,85^ The SEPS process uses a high-yield-stress slurry mechanism, which eliminates the need for building support structures in the printing of complex scaffolds and induces protection of fragile features (e.g., high porosity scaffolds) against process shearing forces. When subject to a force below the yield stress, the material exhibits near solid-like behavior and exerts an elastic force around overhanging components to protect the features against distortion or damage under gravitational force.^59,60,86^ Utilizing SEPS in this study, we have 3D-printed ceramic β-TCP scaffolds with precisely designed internal microarchitectures without the need for supportive structures. Based on previous investigations, pore sizes for bone substitutes are advised to have a minimum pore size of 100 μm, with pore sizes greater than 300 μm recommended to enhance vessel formation, osteocalcin content, and new bone growth.^87−91^ The SEPS-printed β-TCP scaffolds in this study had an average pore size of 410 μm, which is well within the 300–500 μm range reported in the literature for β-TCP-based scaffolds.^92,93^ The SEPS-printed β-TCP scaffolds additionally had a porosity percentage of 44.16%. When compared to porosity percentages reported for SLA-printed components made of hydroxyapatite (38–80%), our β-TCP scaffolds present a lower porosity percentage; however, our β-TCP scaffold pore size and porosity percentage are both within the ranges previously reported for SLA-printed β-TCP scaffolds (28–80%).^92−95^ Taking into consideration that pore size and corresponding scaffold porosity affect the overall mechanical properties of 3D-printed components, we chose scaffold design parameters within the average range of previously reported values in which we would be able to readily fabricate constructs while limiting the potential for part fracture during fabrication or implantation into the rat critical-sized defects. Furthermore, the interconnected porous channels within these β-TCP scaffolds were found to support hBMSC attachment and migration throughout the β-TCP construct. Yet, in this study, as described in previous investigations,^23,38,39,45,46^ the β-TCP scaffolds alone generated limited bone formation in critical-sized calvarial defects. These results further support the need to incorporate strong osteoinductive agents, such as *miR-200c*, into 3D-printed osteoconductive scaffolds.

One way to incorporate osteoinductive biologics into 3D-printed scaffolds is through the use of natural polymeric coatings.^96^ Collagen type-I, a major structural component of bone, is readily available as a hydrogel solution and can easily be incorporated with bioactive agents and coated onto scaffolds.^24^ Hydrogels are often used in drug delivery and act as reservoirs to entrap biomolecules for release *via* diffusion or by degradation of the polymer system.^97^ By providing a mechanism to prolong release of osteoinductive signaling, we may benefit the restoration of larger bone defects for clinical applications. For this investigation, we aimed to prolong the retention of pDNA encoding *miR-200c* to the β-TCP scaffolds and thus increase the duration of osteoinductive signaling by *miR-200c* through coating the β-TCP scaffolds with collagen incorporating pDNA encoding *miR-200c*. Furthermore, the influence of *miR-200c-*incorporated collagen coatings on transfection efficiency of rat and human BMSCs was investigated. From our *in vitro* release studies (Figure 4), we found that the β-TCP scaffolds coated with collagen incorporating pDNA encoding *miR-200c* dramatically improved the retention of pDNA encoding *miR-200c* onto the β-TCP scaffolds compared to noncoated scaffolds soaked in *miR-200c* solution. We observed a burst release of pDNA encoding miR-200c for all scaffolds, regardless of collagen coating, at the 6 h time point. However, scaffolds coated with collagen incorporating *miR-200c* at 5 μg demonstrated a significantly lower percentage of *miR-200c* release throughout the release study observation period compared to noncoated scaffolds. The β-TCP scaffolds coated with collagen incorporating pDNA encoding *miR-200c* at 5 μg demonstrated a lower release rate over the 10 day observation period when compared to noncoated β-TCP scaffolds, where approximately 57% of incorporated *miR-200c* was released from the Coll-*miR-200c* [5 μg] scaffolds by day 10. Collagen-coated scaffolds incorporating *miR-200c* at 5 μg also demonstrated a sustained release function after the first 24 h of release compared to noncoated scaffolds, where noncoated scaffolds quickly released approximately 80% of incorporated *miR-200c* within the first 24 h and approximately 100% of incorporated *miR-200c* by day 6. These data indicate that through use of a collagen coating mechanism, we were able to slow the release of incorporated pDNA encoding *miR-200c* from the β-TCP scaffolds. Such a delivery mechanism may prolong the osteoinductive signaling potential of *miR-200c*-incorporated bone grafts to improve their bone regeneration capacity.

In this study, the osteogenic capacity of naked pDNA encoding *miR-200c* to induce osteogenic differentiation and bone formation from 3D-printed β-TCP scaffolds was assessed under in vitro and *in vivo* conditions. We found that we were able to increase *miR-200c* expression in *miR-200c*-transfected rat BMSCs cultured in both a monolayer cell culture environment and when seeded on β-TCP scaffolds. We additionally observed a significant increase in *miR-200c* expression for both rat and human BMSCs seeded on β-TCP scaffolds coated with collagen incorporating pDNA encoding *miR-200c*. Specifically, collagen-coated β-TCP scaffolds incorporating 5 μg of pDNA encoding *miR-200c* statistically significantly increased *miR-200c* transfection efficiency for both rat and human BMSCs seeded on β-TCP scaffolds across all time points analyzed (Figure 5A–D). Through quantifying osteogenic marker expression *via* qRT-PCR, we found that there was not a significant increase in *Runx2* or *OCN* expression for scaffolds soaked in naked pDNA encoding *miR-200c* without collagen solution *in vitro*. However, these *miR-200c*-alone treated scaffolds displayed significant promotion of bone regeneration in calvarial defects compared to plasmid control, collagen control, and untreated scaffolds when assessed under μCT imaging and through histomorphometric analysis of H&E stained *in vivo* sections. Osteogenic markers were assessed *in vitro* using human BMSCs, while the *in vivo* studies were conducted in rat critical-sized calvarial defects. The differences observed between *miR-200c*-alone treated scaffolds *in vitro* and *in vivo* may be attributed to species differences. For our *in vitro* investigations, we chose to assess osteogenic markers using human BMSCs as the outcomes would be more readily translatable to clinical situations with human patients. Furthermore, from our *in vitro* pDNA release study, we observed a significant burst release profile associated with noncoated *miR-200c*-alone β-TCP scaffolds compared to that with *miR-200c*-collagen-coated samples. These data suggest that *miR-200c* without collagen coating is quickly released into the local environment. Under *in vitro* conditions, this early release may deplete *miR-200c* concentration as it is dispersed into the local culture medium leading to lower cellular uptake of *miR-200c* and ultimately decreased osteogenic marker expression. However, under *in vivo* conditions, *miR-200c* that is quickly released from *miR-200c*-alone treated scaffolds may be readily taken up by cells in the local defect environment, leading to increased bone regeneration in *miR-200c*-treated β-TCP scaffolds.

Moreover, in our current study, we found that incorporation of pDNA encoding *miR-200c* into collagen further increased *in vitro* osteogenic differentiation and *in vivo* bone formation *viamiR-200c* at both low- and high-concentration *miR-200c* compared to plasmid control, collagen control, and untreated β-TCP scaffolds. Incorporation of *miR-200c* into collagen effectively increased the transfection efficiency of *miR-200c* into hBMSCs after 3 days and sustained the overexpression of *miR-200c*. Compared to the β-TCP scaffolds with lyophilized *miR-200c* solution, these results indicated that *miR-200c* incorporated into collagen was quickly taken up and transfected into the cells, which induced more robust osteogenic markers in hBMSCs *in vitro* as evident by enhanced expression of osteogenic markers, including *Runx2*, *OCN*, and *OPG*. Alkaline phosphatase activity was additionally assessed as an osteogenic marker, but a significant increase was not found in our analysis (data not shown). The prolonged release profile of pDNA encoding *miR-200c* from *miR-200c*-collagen-coated scaffolds may further explain the significantly increased expression of osteogenic markers for collagen-coated scaffolds compared to that of noncoated constructs—*miR-200c* concentrations were likely more readily available for cellular uptake when released slowly as opposed to the quick release observed in noncoated scaffolds. Incorporation of *miR-200c* into collagen additionally statistically significantly increases the bone regeneration quantified in the H&E stained sections from our *in vivo* implants, thus effectively demonstrating increased bone regeneration *in vivovia* these *miR-200c-*incorporated scaffolds. These results confirmed the osteogenic capacity of *miR-200c* to regenerate bone tissues and demonstrate the potential of using this hybrid approach combining 3D-printed osteoconductive β-TCP scaffolds with osteoinductive *miR-200c* for bone regeneration in clinical applications.

The results of this study demonstrate that naked pDNA encoding *miR-200c* can efficiently transfect cells to promote osteogenic differentiation and may potentially be used for gene transfection and therapeutic purposes without the limitations and adverse side effects associated with growth factor and viral vector delivery systems. Additionally, the coating of collagen onto β-TCP scaffolds contributed to an upregulation of osteogenic markers in hBMSCs seeded on β-TCP scaffolds with collagen incorporating *miR-200c* at a relatively low dose. Efficient coating of collagen hydrogel substantially improved the transfection of incorporated pDNA encoding *miR-200c*, and the combination of β-TCP scaffolds with collagen/*miR-200c* effectively induced bone regeneration and healed the critical-sized bone defects in rat calvaria. This demonstrates the potential possibility of engineering bone grafts using osteogenic *miR-200c* for the clinical application of bone regeneration. Through this study, we have effectively demonstrated the possibility of combining 3D-printed β-TCP scaffolds with osteogenic *miR-200c* and bioactive collagen for bone regeneration, thus supporting the prospect of fabricating an advanced synthetic bone graft with osteoconductive and -inductive capabilities for clinical application.

## 5 Conclusions

Clinically treating large bone defects is challenging using natural grafts. Traditional scaffold fabrication techniques fall short in producing substitutes that match defect sites with interconnected pores that promote cell migration and nutrient exchange. Furthermore, traditional regenerative approaches often rely on growth factors to promote bone regeneration; however, these agents have been linked to undesired adverse outcomes. In this study, we developed a novel engineered bone graft using a hybrid approach that combines osteoconductive 3D-printed β-TCP scaffolds and osteoinductive *miR-200c* that effectively enhanced bone regeneration. These 3D-printed, microRNA-incorporated grafts may critically impact the development of safe and effective bone substitutes for the clinic.

## References

[ref1] Nauth A.; McKee M. D.; Einhorn T. A.; Watson J. T.; Li R.; Schemitsch E. H. Managing Bone Defects. J. Orthop. Trauma 2011, 25, 462–466. 10.1097/BOT.0b013e318224caf0. 21738065

[ref2] Pobloth A.-M.; Schell H.; Petersen A.; Beierlein K.; Kleber C.; Schmidt-Bleek K.; Duda G. N. Tubular open-porous β-tricalcium phosphate polycaprolactone scaffolds as guiding structure for segmental bone defect regeneration in a novel sheep model. J. Tissue Eng. Regener. Med. 2018, 12, 897–911. 10.1002/term.2446. 28485078

[ref3] Calori G. M.; Mazza E.; Colombo M.; Ripamonti C. The use of bone-graft substitutes in large bone defects: Any specific needs?. Injury 2011, 42, S56–S63. 10.1016/j.injury.2011.06.011. 21752369

[ref4] De Witte T.-M.; Fratila-Apachitei L. E.; Zadpoor A. A.; Peppas N. A. Bone tissue engineering via growth factor delivery: from scaffolds to complex matrices. Regener. Biomater. 2018, 5, 197–211. 10.1093/rb/rby013. PMC6077800 30094059

[ref5] Oryan A.; Alidadi S.; Moshiri A.; Maffulli N. Bone regenerative medicine: classic options, novel strategies, and future directions. J. Orthop. Surg. Res. 2014, 9, 18 10.1186/1749-799x-9-18. 24628910 PMC3995444

[ref6] Baldwin P.; Li D. J.; Auston D. A.; Mir H. S.; Yoon R. S.; Koval K. J. Autograft, Allograft, and Bone Graft Substitutes: Clinical Evidence and Indications for Use in the Setting of Orthopaedic Trauma Surgery. J. Orthop. Trauma 2019, 33, 203–213. 10.1097/bot.0000000000001420. 30633080

[ref7] Salehi S.; Naved B. A.; Grayson W. L. Three-Dimensional Printing Approaches for the Treatment of Critical-Sized Bone Defects; Scrivener Publishing Llc: Beverly, 2017; pp 233–278.

[ref8] Li L.; Zhou G.; Wang Y.; Yang G.; Ding S.; Zhou S. Controlled dual delivery of BMP-2 and dexamethasone by nanoparticle-embedded electrospun nanofibers for the efficient repair of critical-sized rat calvarial defect. Biomaterials 2015, 37, 218–229. 10.1016/j.biomaterials.2014.10.015. 25453952

[ref9] Miszuk J. M.; Xu T.; Yao Q.; Fang F.; Childs J. D.; Hong Z.; Tao J.; Fong H.; Sun H. Functionalization of PCL-3D electrospun nanofibrous scaffolds for improved BMP2-induced bone formation. Appl. Mater. Today 2018, 10, 194–202. 10.1016/j.apmt.2017.12.004. 29577064 PMC5863927

[ref10] Chung Y.-I.; Ahn K.-M.; Jeon S.-H.; Lee S.-Y.; Lee J.-H.; Tae G. Enhanced bone regeneration with BMP-2 loaded functional nanoparticle–hydrogel complex. J. Controlled Release 2007, 121, 91–99. 10.1016/j.jconrel.2007.05.029. 17604871

[ref11] Koh J. T.; Zhao Z.; Wang Z.; Lewis I. S.; Krebsbach P. H.; Franceschi R. T. Combinatorial Gene Therapy with BMP2/7 Enhances Cranial Bone Regeneration. J. Dent. Res. 2008, 87, 845–849. 10.1177/154405910808700906. 18719211 PMC2593032

[ref12] Kim S.; Kim J.; Gajendiran M.; Yoon M.; Hwang M. P.; Wang Y.; Kang B.-J.; Kim K. Enhanced Skull Bone Regeneration by Sustained Release of BMP-2 in Interpenetrating Composite Hydrogels. Biomacromolecules 2018, 19, 4239–4249. 10.1021/acs.biomac.8b01013. 30231204

[ref13] Sawyer A. A.; Song S. J.; Susanto E.; Chuan P.; Lam C. X. F.; Woodruff M. A.; Hutmacher D. W.; Cool S. M. The stimulation of healing within a rat calvarial defect by mPCL–TCP/collagen scaffolds loaded with rhBMP-2. Biomaterials 2009, 30, 2479–2488. 10.1016/j.biomaterials.2008.12.055. 19162318

[ref14] Yang L.; Huang J.; Yang S.; Cui W.; Wang J.; Zhang Y.; Li J.; Guo X. Bone Regeneration Induced by Local Delivery of a Modified PTH-Derived Peptide from Nanohydroxyapatite/Chitosan Coated True Bone Ceramics. ACS Biomater. Sci. Eng. 2018, 4, 3246–3258. 10.1021/acsbiomaterials.7b00780. 33435063

[ref15] Dang M.; Koh A. J.; Jin X.; McCauley L. K.; Ma P. X. Local pulsatile PTH delivery regenerates bone defects via enhanced bone remodeling in a cell-free scaffold. Biomaterials 2017, 114, 1–9. 10.1016/j.biomaterials.2016.10.049. 27835763 PMC5125900

[ref16] Linh N. T. B.; Abueva C. D. G.; Jang D.-W.; Lee B.-T. Collagen and bone morphogenetic protein-2 functionalized hydroxyapatite scaffolds induce osteogenic differentiation in human adipose-derived stem cells. J. Biomed. Mater. Res., Part B 2020, 1363–1371. 10.1002/jbm.b.34485. 31574204

[ref17] Alluri R.; Song X.; Bougioukli S.; Pannell W.; Vakhshori V.; Sugiyama O.; Tang A.; Park S. H.; Chen Y.; Lieberman J. R. Regional gene therapy with 3D printed scaffolds to heal critical sized bone defects in a rat model. J. Biomed. Mater. Res., Part A 2019, 2174–2182. 10.1002/jbm.a.36727. PMC7699135 31112357

[ref18] Chen G. H.; Sun Y.; Lu F. Z.; Jiang A. L.; Subedi D.; Kong P. Y.; Wang X. Y.; Yu T. L.; Chi H.; Song C. C.; Liu K. Y.; Qi P. F.; Yan J. L.; Ji Y. A three-dimensional (3D) printed biomimetic hierarchical scaffold with a covalent modular release system for osteogenesis. Mater. Sci. Eng., C 2019, 104, 109842 10.1016/j.msec.2019.109842. 31500042

[ref19] Mitchell A. C.; Briquez P. S.; Hubbell J. A.; Cochran J. R. Engineering growth factors for regenerative medicine applications. Acta Biomater. 2016, 30, 1–12. 10.1016/j.actbio.2015.11.007. 26555377 PMC6067679

[ref20] Weisgerber D. W.; Caliari S. R.; Harley B. A. C. Mineralized collagen scaffolds induce hMSC osteogenesis and matrix remodeling. Biomater. Sci. 2015, 3, 533–542. 10.1039/c4bm00397g. 25937924 PMC4412464

[ref21] Min Z.; Zhao S. C.; Xin C.; Zhu Y. F.; Zhang C. Q. 3D-printed dimethyloxallyl glycine delivery scaffolds to improve angiogenesis and osteogenesis. Biomater. Sci. 2015, 3, 1236–1244. 10.1039/c5bm00132c. 26222039

[ref22] Balmayor E. R.; van Griensven M. Gene therapy for bone engineering. Front. Bioeng. Biotechnol. 2015, 3, 9 10.3389/fbioe.2015.00009. 25699253 PMC4313589

[ref23] Sawada K.; Nakahara K.; Haga-Tsujimura M.; Iizuka T.; Fujioka-Kobayashi M.; Igarashi K.; Saulacic N. Comparison of three block bone substitutes for bone regeneration: long-term observation in the beagle dog. Odontology 2018, 106, 398–407. 10.1007/s10266-018-0352-7. 29557992

[ref24] Santo V. E.; Gomes M. E.; Mano J. F.; Reis R. L. Controlled Release Strategies for Bone, Cartilage, and Osteochondral Engineering-Part I: Recapitulation of Native Tissue Healing and Variables for the Design of Delivery Systems. Tissue Eng., Part B 2013, 19, 308–326. 10.1089/ten.teb.2012.0138. PMC3690094 23268651

[ref25] Amini A. R.; Laurencin C. T.; Nukavarapu S. P. Bone tissue engineering: recent advances and challenges. Crit. Rev. Biomed. Eng. 2012, 40, 363–408. 10.1615/critrevbiomedeng.v40.i5.10. 23339648 PMC3766369

[ref26] Zafar M. J.; Zhu D.; Zhang Z. 3D Printing of Bioceramics for Bone Tissue Engineering. Materials 2019, 12, 3361 10.3390/ma12203361. PMC6829398 31618857

[ref27] Brie J.; Chartier T.; Chaput C.; Delage C.; Pradeau B.; Caire F.; Boncoeur M.-P.; Moreau J.-J. A new custom made bioceramic implant for the repair of large and complex craniofacial bone defects. J. Cranio-Maxillofac. Surg. 2013, 41, 403–407. 10.1016/j.jcms.2012.11.005. 23218977

[ref28] Wubneh A.; Tsekoura E. K.; Ayranci C.; Uludag H. Current state of fabrication technologies and materials for bone tissue engineering. Acta Biomater. 2018, 80, 1–30. 10.1016/j.actbio.2018.09.031. 30248515

[ref29] Bittner S. M.; Smith B. T.; Diaz-Gomez L.; Hudgins C. D.; Melchiorri A. J.; Scott D. W.; Fisher J. P.; Mikos A. G. Fabrication and mechanical characterization of 3D printed vertical uniform and gradient scaffolds for bone and osteochondral tissue engineering. Acta Biomater. 2019, 90, 37–48. 10.1016/j.actbio.2019.03.041. 30905862 PMC6744258

[ref30] Chu T. M. G.; Orton D. G.; Hollister S. J.; Feinberg S. E.; Halloran J. W. Mechanical and in vivo performance of hydroxyapatite implants with controlled architectures. Biomaterials 2002, 23, 1283–1293. 10.1016/S0142-9612(01)00243-5. 11808536

[ref31] Oryan A.; Baghaban Eslaminejad M.; Kamali A.; Hosseini S.; Moshiri A.; Baharvand H. Mesenchymal stem cells seeded onto tissue-engineered osteoinductive scaffolds enhance the healing process of critical-sized radial bone defects in rat. Cell Tissue Res. 2018, 374, 63–81. 10.1007/s00441-018-2837-7. 29717356

[ref32] Sultana N.; Hassan M. I.; Lim M. M. Scaffold Fabrication Protocols. In Composite Synthetic Scaffolds for Tissue Engineering and Regenerative Medicine; Springer International Publishing: Cham, 2015; pp 13–24.

[ref33] Cho Y. S.; Quan M.; Lee S.-H.; Hong M. W.; Kim Y. Y.; Cho Y.-S. Assessment of osteogenesis for 3D-printed polycaprolactone/hydroxyapatite composite scaffold with enhanced exposure of hydroxyapatite using rat calvarial defect model. Compos. Sci. Technol. 2019, 184, 107844 10.1016/j.compscitech.2019.107844.

[ref34] Sachlos E.; Czernuszka J. T. Making tissue engineering scaffolds work. Review: the application of solid freeform fabrication technology to the production of tissue engineering scaffolds. Eur. Cells Mater. 2003, 5, 29–39. 10.22203/eCM.v005a03. 14562270

[ref35] Seitz H.; Wolfgang R.; Stephan I.; Barbara L.; Carsten T. Three-dimensional printing of porous ceramic scaffolds for bone tissue engineering. J. Biomed. Mater. Res., Part B 2005, 74B, 782–788. 10.1002/jbm.b.30291. 15981173

[ref36] Warnke P. H.; Seitz H.; Warnke F.; Becker S. T.; Sivananthan S.; Sherry E.; Liu Q.; Wiltfang J.; Douglas T. Ceramic scaffolds produced by computer-assisted 3D printing and sintering: Characterization and biocompatibility investigations. J. Biomed. Mater. Res., Part B 2010, 93B, 212–217. 10.1002/jbm.b.31577. 20091914

[ref37] Tarafder S.; Balla V. K.; Davies N. M.; Bandyopadhyay A.; Bose S. Microwave-sintered 3D printed tricalcium phosphate scaffolds for bone tissue engineering. J. Tissue Eng. Regener. Med. 2013, 7, 631–641. 10.1002/term.555. PMC4182013 22396130

[ref38] Liu B.; Lun D. X. Current application of β-tricalcium phosphate composites in orthopaedics. Orthop. Surg. 2012, 4, 139–144. 10.1111/j.1757-7861.2012.00189.x. 22927147 PMC6583186

[ref39] Rh Owen G.; Dard M.; Larjava H. Hydoxyapatite/beta-tricalcium phosphate biphasic ceramics as regenerative material for the repair of complex bone defects. J. Biomed. Mater. Res., Part B 2018, 106, 2493–2512. 10.1002/jbm.b.34049. 29266701

[ref40] Liu H.; Yazici H.; Ergun C.; Webster T. J.; Bermek H. An in vitro evaluation of the Ca/P ratio for the cytocompatibility of nano-to-micron particulate calcium phosphates for bone regeneration. Acta Biomater. 2008, 4, 1472–1479. 10.1016/j.actbio.2008.02.025. 18394980

[ref41] Wang G. C.; Lu Z. F.; Zreiqat H. Bioceramics for Skeletal Bone Regeneration. In Bone Substitute Biomaterials; Mallick K., Ed.; Woodhead Publishing, 2014; pp 180–216.

[ref42] Bai X.; Gao M.; Syed S.; Zhuang J.; Xu X.; Zhang X.-Q. Bioactive hydrogels for bone regeneration. Bioact. Mater. 2018, 3, 401–417. 10.1016/j.bioactmat.2018.05.006. 30003179 PMC6038268

[ref43] Huang B.; Caetano G.; Vyas C.; Blaker J. J.; Diver C.; Bártolo P. Polymer-Ceramic Composite Scaffolds: The Effect of Hydroxyapatite and β-tri-Calcium Phosphate. Materials 2018, 11, 129 10.3390/ma11010129. PMC5793627 29342890

[ref44] Dong L.; Wang S.-J.; Zhao X.-R.; Zhu Y.-F.; Yu J.-K. 3D- Printed Poly(ε-caprolactone) Scaffold Integrated with Cell-laden Chitosan Hydrogels for Bone Tissue Engineering. Sci. Rep. 2017, 7, 13412 10.1038/s41598-017-13838-7. 29042614 PMC5645328

[ref45] Tarafder S.; Davies N. M.; Bandyopadhyay A.; Bose S. 3D printed tricalcium phosphate bone tissue engineering scaffolds: effect of SrO and MgO doping on in vivo osteogenesis in a rat distal femoral defect model. Biomater. Sci. 2013, 1, 1250–1259. 10.1039/c3bm60132c. 24729867 PMC3979641

[ref46] Cao H.; Kuboyama N. A biodegradable porous composite scaffold of PGA/β-TCP for bone tissue engineering. Bone 2010, 46, 386–395. 10.1016/j.bone.2009.09.031. 19800045

[ref47] Yuan H.; Fernandes H.; Habibovic P.; de Boer J.; Barradas A. M. C.; de Ruiter A.; Walsh W. R.; van Blitterswijk C. A.; de Bruijn J. D. Osteoinductive ceramics as a synthetic alternative to autologous bone grafting. Proc. Natl. Acad. Sci. U.S.A. 2010, 107, 13614–13619. 10.1073/pnas.1003600107. 20643969 PMC2922269

[ref48] Peng S. P.; Gao D.; Gao C. D.; Wei P. P.; Niu M.; Shuai C. J. MicroRNAs regulate signaling pathways in osteogenic differentiation of mesenchymal stem cells (Review). Mol. Med. Rep. 2016, 14, 623–629. 10.3892/mmr.2016.5335. 27222009 PMC4918597

[ref49] Vallejo D. M.; Caparros E.; Dominguez M. Targeting Notch signalling by the conserved miR-8/200 microRNA family in development and cancer cells. EMBO J. 2011, 30, 756–769. 10.1038/emboj.2010.358. 21224847 PMC3041954

[ref50] Hill L.; Browne G.; Tulchinsky E. ZEB/miR-200 feedback loop: at the crossroads of signal transduction in cancer. Int. J. Cancer 2013, 132, 745–754. 10.1002/ijc.27708. 22753312

[ref51] Katoh Y.; Katoh M. Hedgehog signaling, epithelial-to-mesenchymal transition and miRNA (Review). Int. J. Mol. Med. 2008, 22, 271–275. 10.3892/ijmm_00000019. 18698484

[ref52] Humphries B.; Yang C. The microRNA-200 family: small molecules with novel roles in cancer development, progression and therapy. Oncotarget 2015, 6, 6472–6498. 10.18632/oncotarget.3052. 25762624 PMC4466628

[ref53] Akkouch A.; Eliason S.; Sweat M. E.; Romero-Bustillos M.; Zhu M.; Qian F.; Amendt B. A.; Hong L. Enhancement of MicroRNA-200c on Osteogenic Differentiation and Bone Regeneration by Targeting Sox2-Mediated Wnt Signaling and Klf4. Hum. Gene Ther. 2019, 30, 1405–1418. 10.1089/hum.2019.019. 31288577 PMC6854517

[ref54] Akkouch A.; Zhu M.; Romero-Bustillos M.; Eliason S.; Qian F.; Salem A. K.; Amendt B. A.; Hong L. MicroRNA-200c Attenuates Periodontitis by Modulating Proinflammatory and Osteoclastogenic Mediators. Stem Cells Dev. 2019, 28, 1026–1036. 10.1089/scd.2019.0027. 31017046 PMC6661922

[ref55] Chuang T. D.; Khorram O. miR-200c Regulates IL8 Expression by Targeting IKBKB: A Potential Mediator of Inflammation in Leiomyoma Pathogenesis. PLoS One 2014, 9, e95370 10.1371/journal.pone.0095370. 24755559 PMC3995706

[ref56] Hong L.; Sharp T.; Khorsand B.; Fischer C.; Eliason S.; Salem A.; Akkouch A.; Brogden K.; Amendt B. A. MicroRNA-200c Represses IL-6, IL-8, and CCL-5 Expression and Enhances Osteogenic Differentiation. PLoS One 2016, 11, e0160915 10.1371/journal.pone.0160915. 27529418 PMC4987006

[ref57] Cao H.; Jheon A.; Li X.; Sun Z.; Wang J.; Florez S.; Zhang Z.; McManus M. T.; Klein O. D.; Amendt B. A. The Pitx2:miR-200c/141:noggin pathway regulates Bmp signaling and ameloblast differentiation. Development 2013, 140, 3348–3359. 10.1242/dev.089193. 23863486 PMC3737717

[ref58] Fajardo M.; Liu C.-J.; Egol K. Levels of expression for BMP-7 and several BMP antagonists may play an integral role in a fracture nonunion: a pilot study. Clin. Orthop. Relat. Res. 2009, 467, 3071–3078. 10.1007/s11999-009-0981-9. 19597895 PMC2772945

[ref59] He L.; Fei F.; Wang W.; Song X. Support-Free Ceramic Stereolithography of Complex Overhanging Structures Based on an Elasto-viscoplastic Suspension Feedstock. ACS Appl. Mater. Interfaces 2019, 11, 18849–18857. 10.1021/acsami.9b04205. 31059219

[ref60] He L.; Song X. Supportability of a High-Yield-Stress Slurry in a New Stereolithography-Based Ceramic Fabrication Process. JOM 2018, 70, 407–412. 10.1007/s11837-017-2657-3.

[ref61] Li X.; Zhang Y.; Qi G. Evaluation of isolation methods and culture conditions for rat bone marrow mesenchymal stem cells. Cytotechnology 2013, 65, 323–334. 10.1007/s10616-012-9497-3. 23011741 PMC3597169

[ref62] Tang D.; Tare R. S.; Yang L.-Y.; Williams D. F.; Ou K.-L.; Oreffo R. O. C. Biofabrication of bone tissue: approaches, challenges and translation for bone regeneration. Biomaterials 2016, 83, 363–382. 10.1016/j.biomaterials.2016.01.024. 26803405

[ref63] Florencio-Silva R.; da Silva Sasso G. R.; Sasso-Cerri E.; Simões M. J.; Cerri P. S. Biology of Bone Tissue: Structure, Function, and Factors That Influence Bone Cells. BioMed Res. Int. 2015, 2015, 1–17. 10.1155/2015/421746. PMC4515490 26247020

[ref64] Hannink G.; Arts J. J. C. Bioresorbability, porosity and mechanical strength of bone substitutes: What is optimal for bone regeneration?. Injury 2011, 42, S22–S25. 10.1016/j.injury.2011.06.008. 21714966

[ref65] Yin H.; Kanasty R. L.; Eltoukhy A. A.; Vegas A. J.; Dorkin J. R.; Anderson D. G. Non-viral vectors for gene-based therapy. Nat. Rev. Genet. 2014, 15, 541–555. 10.1038/nrg3763. 25022906

[ref66] Sheikh Z.; Javaid M. A.; Hamdan N.; Hashmi R. Bone Regeneration Using Bone Morphogenetic Proteins and Various Biomaterial Carriers. Materials 2015, 8, 1778–1816. 10.3390/ma8041778. 28788032 PMC5507058

[ref67] Lee K.; Silva E. A.; Mooney D. J. Growth factor delivery-based tissue engineering: general approaches and a review of recent developments. J. R. Soc. Interface 2011, 8, 153–170. 10.1098/rsif.2010.0223. 20719768 PMC3033020

[ref68] Carlos M.; Dario P.; Federica C.; Emo C. Additive manufacturing techniques for the production of tissue engineering constructs. J. Tissue Eng. Regener. Med. 2015, 9, 174–190. 10.1002/term.1635. 23172792

[ref69] Zein I.; Hutmacher D. W.; Tan K. C.; Teoh S. H. Fused deposition modeling of novel scaffold architectures for tissue engineering applications. Biomaterials 2002, 23, 1169–1185. 10.1016/S0142-9612(01)00232-0. 11791921

[ref70] Korpela J.; Kokkari A.; Korhonen H.; Malin M.; Närhi T.; Seppälä J. Biodegradable and bioactive porous scaffold structures prepared using fused deposition modeling. J. Biomed. Mater. Res., Part B 2013, 101B, 610–619. 10.1002/jbm.b.32863. 23281260

[ref71] An J.; Teoh J. E. M.; Suntornnond R.; Chua C. K. Design and 3D Printing of Scaffolds and Tissues. Engineering 2015, 1, 261–268. 10.15302/J-ENG-2015061.

[ref72] Elomaa L.; Teixeira S.; Hakala R.; Korhonen H.; Grijpma D. W.; Seppälä J. V. Preparation of poly(ε-caprolactone)-based tissue engineering scaffolds by stereolithography. Acta Biomater. 2011, 7, 3850–3856. 10.1016/j.actbio.2011.06.039. 21763796

[ref73] Do A.-V.; Khorsand B.; Geary S. M.; Salem A. K. 3D Printing of Scaffolds for Tissue Regeneration Applications. Adv. Healthcare Mater. 2015, 4, 1742–1762. 10.1002/adhm.201500168. PMC4597933 26097108

[ref74] Buyuksungur S.; Tanir T. E.; Buyuksungur A.; Bektas E. I.; Kose G. T.; Yucel D.; Beyzadeoglu T.; Cetinkaya E.; Yenigun C.; Tonuk E.; Hasirci V.; Hasirci N. 3D printed poly(epsilon-caprolactone) scaffolds modified with hydroxyapatite and poly(propylene fumarate) and their effects on the healing of rabbit femur defects. Biomater. Sci. 2017, 5, 2144–2158. 10.1039/c7bm00514h. 28880313

[ref75] Fonseca D. R.; Sobreiro-Almeida R.; Sol P. C.; Neves N. M. Development of non-orthogonal 3D-printed scaffolds to enhance their osteogenic performance. Biomater. Sci. 2018, 6, 1569–1579. 10.1039/c8bm00073e. 29708246

[ref76] Jammalamadaka U.; Tappa K. Recent Advances in Biomaterials for 3D Printing and Tissue Engineering. J. Funct. Biomater. 2018, 9, 22 10.3390/jfb9010022. PMC5872108 29494503

[ref77] Seidenstuecker M.; Kerr L.; Bernstein A.; Mayr H. O.; Suedkamp N. P.; Gadow R.; Krieg P.; Hernandez Latorre S.; Thomann R.; Syrowatka F.; Esslinger S. 3D Powder Printed Bioglass and β-Tricalcium Phosphate Bone Scaffolds. Materials 2018, 11, 13 10.3390/ma11010013. PMC5793511 29271932

[ref78] Zafar M. J.; Zhu D. B.; Zhang Z. Y. 3D Printing of Bioceramics for Bone Tissue Engineering. Materials 2019, 12, 3361 10.3390/ma12203361. PMC6829398 31618857

[ref79] Bose S.; Vahabzadeh S.; Bandyopadhyay A. Bone tissue engineering using 3D printing. Mater. Today 2013, 16, 496–504. 10.1016/j.mattod.2013.11.017.

[ref80] Zhang A. P.; Xin Q.; Pranav S.; C H. K.; W L. J.; Shaochen C.; Sailing H. Rapid Fabrication of Complex 3D Extracellular Microenvironments by Dynamic Optical Projection Stereolithography. Adv. Mater. 2012, 24, 4266–4270. 10.1002/adma.201202024. 22786787 PMC3789064

[ref81] Gauvin R.; Chen Y.-C.; Lee J. W.; Soman P.; Zorlutuna P.; Nichol J. W.; Bae H.; Chen S.; Khademhosseini A. Microfabrication of complex porous tissue engineering scaffolds using 3D projection stereolithography. Biomaterials 2012, 33, 3824–3834. 10.1016/j.biomaterials.2012.01.048. 22365811 PMC3766354

[ref82] Melchels F. P. W.; Feijen J.; Grijpma D. W. A review on stereolithography and its applications in biomedical engineering. Biomaterials 2010, 31, 6121–6130. 10.1016/j.biomaterials.2010.04.050. 20478613

[ref83] van Tienderen G. S.; Berthel M.; Yue Z.; Cook M.; Liu X.; Beirne S.; Wallace G. G. Advanced fabrication approaches to controlled delivery systems for epilepsy treatment. Expert Opin. Drug Delivery 2018, 15, 915–925. 10.1080/17425247.2018.1517745. 30169981

[ref84] Jiang J.; Xu X.; Stringer J. Support Structures for Additive Manufacturing: A Review. J. Manuf. Mater. Process. 2018, 2, 64 10.3390/jmmp2040064.

[ref85] Chen Z.; Qian X.; Song X.; Jiang Q.; Huang R.; Yang Y.; Li R.; Shung K.; Chen Y.; Zhou Q. Three-Dimensional Printed Piezoelectric Array for Improving Acoustic Field and Spatial Resolution in Medical Ultrasonic Imaging. Micromachines 2019, 10, 170 10.3390/mi10030170. PMC6471007 30823480

[ref86] Song X.; Chen Y.; Lee T. W.; Wu S.; Cheng L. Ceramic fabrication using Mask-Image-Projection-based Stereolithography integrated with tape-casting. J. Manuf. Processes 2015, 20, 456–464. 10.1016/j.jmapro.2015.06.022.

[ref87] Kuboki Y.; Jin Q.; Takita H. Geometry of Carriers Controlling Phenotypic Expression in BMP-Induced Osteogenesis and Chondrogenesis. J. Bone Jt. Surg., Am. Vol. 2001, 83, S1–105. 10.2106/00004623-200100002-00005. 11314788

[ref88] Tsuruga E.; Takita H.; Itoh H.; Wakisaka Y.; Kuboki Y. Pore Size of Porous Hydroxyapatite as the Cell-Substratum Controls BMP-Induced Osteogenesis1. J. Biochem. 1997, 121, 317–324. 10.1093/oxfordjournals.jbchem.a021589. 9089406

[ref89] Dong J.; Kojima H.; Uemura T.; Kikuchi M.; Tateishi T.; Tanaka J. In vivo evaluation of a novel porous hydroxyapatite to sustain osteogenesis of transplanted bone marrow-derived osteoblastic cells. J. Biomed. Mater. Res. 2001, 57, 208–216. 10.1002/1097-4636(200111)57:2<208::AID-JBM1160>3.0.CO;2-N. 11484183

[ref90] Hulbert S. F.; Young F. A.; Mathews R. S.; Klawitter J. J.; Talbert C. D.; Stelling F. H. Potential of ceramic materials as permanently implantable skeletal prostheses. J. Biomed. Mater. Res. 1970, 4, 433–456. 10.1002/jbm.820040309. 5469185

[ref91] Dellinger J. G.; Wojtowicz A. M.; Jamison R. D. Effects of degradation and porosity on the load bearing properties of model hydroxyapatite bone scaffolds. J. Biomed. Mater. Res., Part A 2006, 77A, 563–571. 10.1002/jbm.a.30658. 16498598

[ref92] Wagoner Johnson A. J.; Herschler B. A. A review of the mechanical behavior of CaP and CaP/polymer composites for applications in bone replacement and repair. Acta Biomater. 2011, 7, 16–30. 10.1016/j.actbio.2010.07.012. 20655397

[ref93] Karageorgiou V.; Kaplan D. Porosity of 3D biomaterial scaffolds and osteogenesis. Biomaterials 2005, 26, 5474–5491. 10.1016/j.biomaterials.2005.02.002. 15860204

[ref94] Bohner M.; Santoni B. L. G.; Döbelin N. β-tricalcium phosphate for bone substitution: Synthesis and properties. Acta Biomater. 2020, 113, 23–41. 10.1016/j.actbio.2020.06.022. 32565369

[ref95] Bouler J. M.; Pilet P.; Gauthier O.; Verron E. Biphasic calcium phosphate ceramics for bone reconstruction: A review of biological response. Acta Biomater. 2017, 53, 1–12. 10.1016/j.actbio.2017.01.076. 28159720

[ref96] Elzoghby A. O. Gelatin-based nanoparticles as drug and gene delivery systems: Reviewing three decades of research. J. Controlled Release 2013, 172, 1075–1091. 10.1016/j.jconrel.2013.09.019. 24096021

[ref97] Elangovan S.; Gajendrareddy P.; Ravindran S.; Salem A. K. Emerging local delivery strategies to enhance bone regeneration. Biomed. Mater. 2020, 15, 062001 10.1088/1748-605X/aba446. 32647095 PMC10148649

